# Differential processing of a chemosensory cue across life stages sharing the same valence state in *Caenorhabditis elegans*

**DOI:** 10.1073/pnas.2218023120

**Published:** 2023-05-01

**Authors:** Navonil Banerjee, Pei-Yin Shih, Elisa J. Rojas Palato, Paul W. Sternberg, Elissa A. Hallem

**Affiliations:** ^a^Department of Microbiology, Immunology, and Molecular Genetics, University of California, Los Angeles, CA 90095; ^b^Molecular Biology Institute, University of California, Los Angeles, CA 90095; ^c^Division of Biology and Biological Engineering, California Institute of Technology, Pasadena, CA 91125

**Keywords:** behavior, neural circuits, chemotaxis, carbon dioxide, dauer larva

## Abstract

Many chemosensory cues evoke responses of the same valence under widely varying physiological conditions. It remains unclear whether similar or distinct neural mechanisms are involved in the detection and processing of such chemosensory cues across contexts. We show that in *Caenorhabditis elegans*, a chemosensory cue is processed by distinct neural mechanisms at two different life stages that share the same valence state. Both starved adults and dauer larvae are attracted to carbon dioxide (CO_2_), but CO_2_ evokes different patterns of neural activity and different motor outputs at the two life stages. Moreover, the same interneuron within the CO_2_ microcircuit plays a different role in driving CO_2_-evoked motor output at the two life stages. The dauer-specific patterns of CO_2_-evoked activity in this interneuron require a dauer-specific gap junction complex and insulin signaling. Our results demonstrate that functionally distinct microcircuits are engaged in response to a chemosensory cue that triggers the same valence state at different life stages, revealing an unexpected complexity to chemosensory processing.

## Significance

Animals frequently show the same preference toward a chemosensory cue under widely varying external and internal conditions. Whether such chemosensory cues involve similar neural mechanisms across conditions is unclear. Here, we show that carbon dioxide (CO_2_) is processed through distinct neural mechanisms in *C. elegans* at two different life stages that show the same preference for CO_2_. These mechanistic differences are manifested in altered CO_2_-evoked neuronal activity and motor output. A life stage–specific change in neural connectivity and insulin signaling contribute to these circuit differences by modulating the functional properties of an interneuron. We demonstrate that distinct neural mechanisms may underlie the same preference for a chemosensory cue and highlight the importance of physiological context in understanding chemosensory behaviors.

Chemosensation is crucial for animals to successfully navigate their environments and accomplish essential goal-directed behaviors such as locating food, searching for mates, and escaping predators (1). As a result, many chemosensory behaviors and their underlying mechanisms are highly flexible and can be modulated by an animal’s internal physiological state (2–7). For example, the same chemosensory cue can evoke distinct brain-wide activity dynamics in thirsty vs. sated mice (8) and distinct neural activity in fed vs. starved *Drosophila* larvae (9). In some cases, changes in internal physiological state result in a switch in chemosensory valence, i.e., whether the chemosensory cue is perceived as attractive or aversive (10–13). In contrast, other chemosensory cues can evoke responses of the same valence under very different physiological conditions (14–16). How the same valence state is maintained given the constraints posed by changes in internal physiology on chemosensory processing remains poorly understood.

We explore these mechanisms using the chemosensory responses of *Caenorhabditis elegans* to carbon dioxide (CO_2_). *C. elegans* has a small nervous system with a well-characterized connectome (17–19). In addition, *C. elegans* responds robustly to a diverse array of chemosensory cues, including CO_2_ (20). CO_2_ is an ambiguous cue for *C. elegans*, as elevated CO_2_ levels in its natural habitat may signal food, predators, pathogens, or conspecifics (20, 21). Accordingly, *C. elegans* shows flexible responses to CO_2_ such that CO_2_ can be either attractive or repulsive depending on immediate context, prior experience, and life stage (22–28). For example, while well-fed *C. elegans* adults are repelled by CO_2_, starvation results in a shift in CO_2_ response valence such that starved adults are attracted to CO_2_ (22, 23, 28).

Under adverse environmental conditions such as absence of food, high temperature, and overcrowding, *C. elegans* enters the developmentally arrested dauer larval stage (29, 30). Dauer entry is accompanied by a dramatic reprogramming of internal physiology that promotes developmental arrest and prolonged survival under unfavorable conditions (31). Like starved adults, dauer larvae are robustly attracted to CO_2_ despite their dramatically different physiology (24). Although the neural mechanisms responsible for the detection and processing of CO_2_ have been partly elucidated in starved adults (28), the dauer CO_2_ circuit had not been investigated.

Here, we show that distinct neural mechanisms are involved in the detection and processing of CO_2_ in dauers and starved adults. At a circuit level, we observe differences in the functional properties of the CO_2_-detecting BAG neurons as well as downstream interneurons. The BAG sensory neurons show reduced CO_2_-evoked calcium responses in dauers compared to starved adults. In addition, the RIG, AIB, AVE, and AIY interneurons respond differently to CO_2_ at the two life stages. A dauer-specific gap junction complex and insulin signaling contribute to the dauer-specific response properties of the AIB interneurons. Differences in the functional CO_2_ microcircuit are reflected in distinct locomotory patterns that are triggered by acute CO_2_ exposure at the two life stages. In addition, the AIB interneurons have opposing effects on CO_2_-evoked movement in starved adults vs. dauers: AIB promotes CO_2_-evoked reversals in starved adults but inhibits CO_2_-evoked reversals in dauers. Together, our findings illustrate that functionally distinct microcircuits are engaged by a chemosensory cue at two different life stages that share the same valence state, highlighting the importance of physiological context in understanding chemosensory behaviors.

## Results

### The BAG Neurons of Dauer Larvae Show Reduced CO_2_-Evoked Activity.

**Fig. 1.**
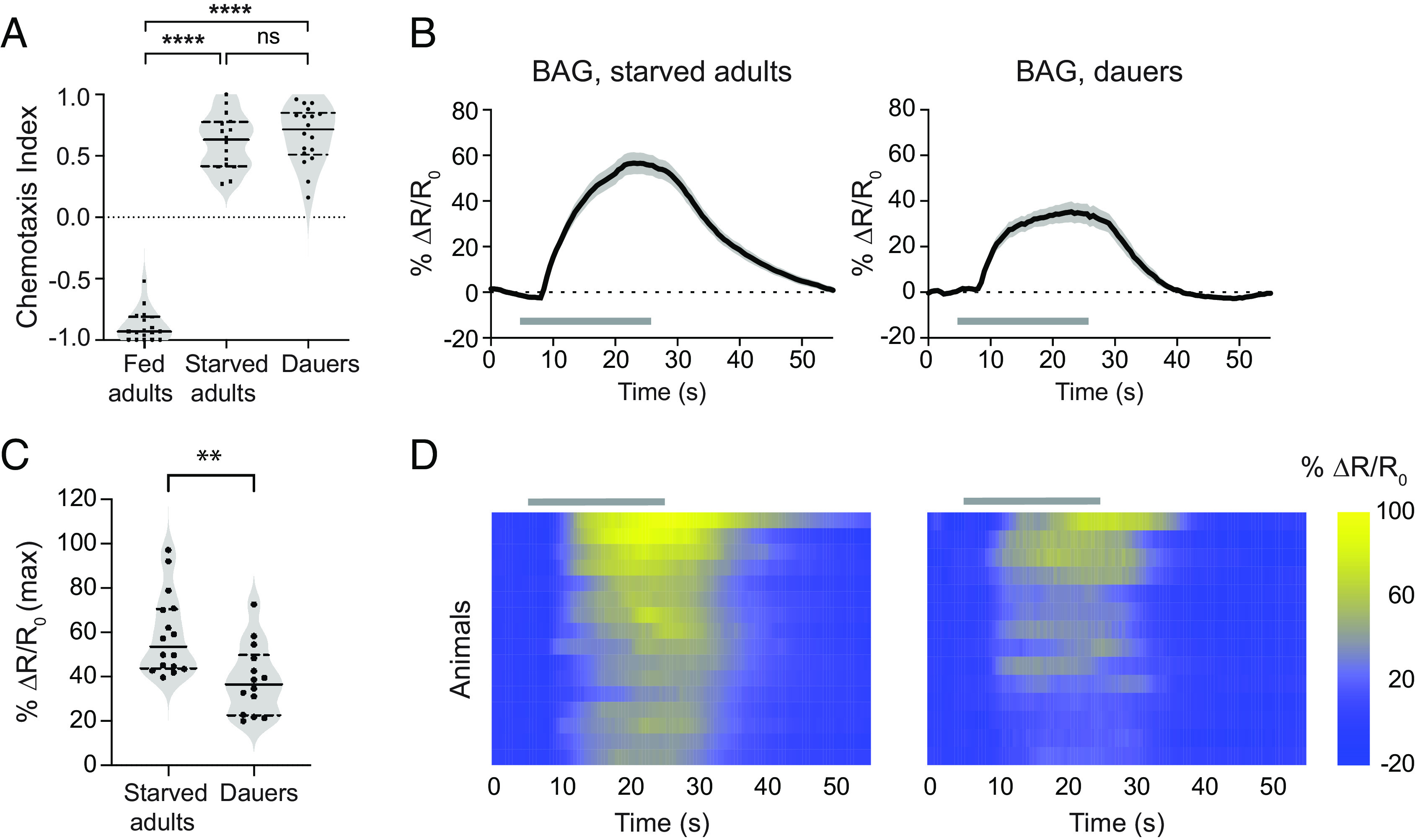
The BAG sensory neurons show reduced calcium responses in dauers. (*A*) Well-fed adults are repelled by CO_2_, whereas starved adults and dauers are attracted to CO_2_. n = 16 to 18 trials per life stage and condition. ****P < 0.0001, ns = not significant (*P *> 0.9999), Kruskal–Wallis test with Dunn’s posttest. (*B*) Calcium responses in the BAG neurons of starved adults and dauers. Solid lines indicate mean calcium responses and shadings indicate SEM. Gray bars indicate timing and duration of the CO_2_ pulse. Calcium responses were measured using the ratiometric calcium indicator yellow cameleon YC3.60. Responses are to 15% CO_2_. (*C*) Quantification of the maximum responses of BAG in starved adults and dauers. Each data point represents the response of a single animal. Solid lines in violin plots show medians and dotted lines show interquartile ranges. **P < 0.01, Welch’s *t* test. n = 14 to 16 animals per life stage. Responses are to 15% CO_2_. (*D*) Heatmaps of BAG calcium responses. Each row represents the response of an individual animal. Response magnitudes in the heatmaps are color-coded according to the scale (% ΔR/R_0_) shown to the right. Responses are ordered by hierarchical cluster analysis. Gray bars indicate the timing and duration of the CO_2_ pulse.

Well-fed *C. elegans* adults are repelled by CO_2_, whereas both starved adults and dauer larvae are attracted to CO_2_ (Fig. 1*A*) (24, 27, 28). Does the altered physiology of dauers modify the functional properties of neurons within the CO_2_ microcircuit? To address this question, we first monitored the CO_2_-evoked calcium activity of the primary CO_2_-detecting BAG neurons, which are required for CO_2_ attraction in both starved adults and dauers (22, 24, 28, 32). Using the genetically encoded ratiometric calcium indicator yellow cameleon YC3.60, we found that the BAG neurons of both starved adults and dauers are activated by CO_2_ (Fig. 1 *B*–*D*). However, the BAG neurons of dauers show a reduced response to CO_2_ relative to that of starved adults (Fig. 1*C*). Thus, functional differences in the CO_2_ microcircuit between starved adults and dauers occur at the sensory neuron level.

To test whether the reduced CO_2_-evoked calcium activity of BAG in dauers results from decreased expression of the putative CO_2_ receptor GCY-9 (32, 33), we used a strain expressing green fluorescent protein (GFP) under the control of the *gcy-9* promoter and quantified fluorescent intensities in BAG neuron cell bodies at the two life stages. We did not observe a noticeable difference in GFP expression in the BAG cell bodies of starved adults vs. dauers (*SI Appendix*, Fig. S1 *A* and *B*), suggesting that the reduced response of dauer BAG neurons does not reflect decreased CO_2_ receptor expression. It also does not reflect altered expression of the calcium indicator (*SI Appendix*, Fig. S1 *C* and *D*). The reduced response of dauer BAG neurons to CO_2_ could result from reduced diffusion of CO_2_ through the thicker cuticle of dauers and/or reduced sensitivity of dauer BAG neurons through a mechanism independent of *gcy-9* expression.

### CO_2_ Microcircuit Interneurons Show Distinct Patterns of CO_2_-Evoked Activity in Dauers.

**Fig. 2.**
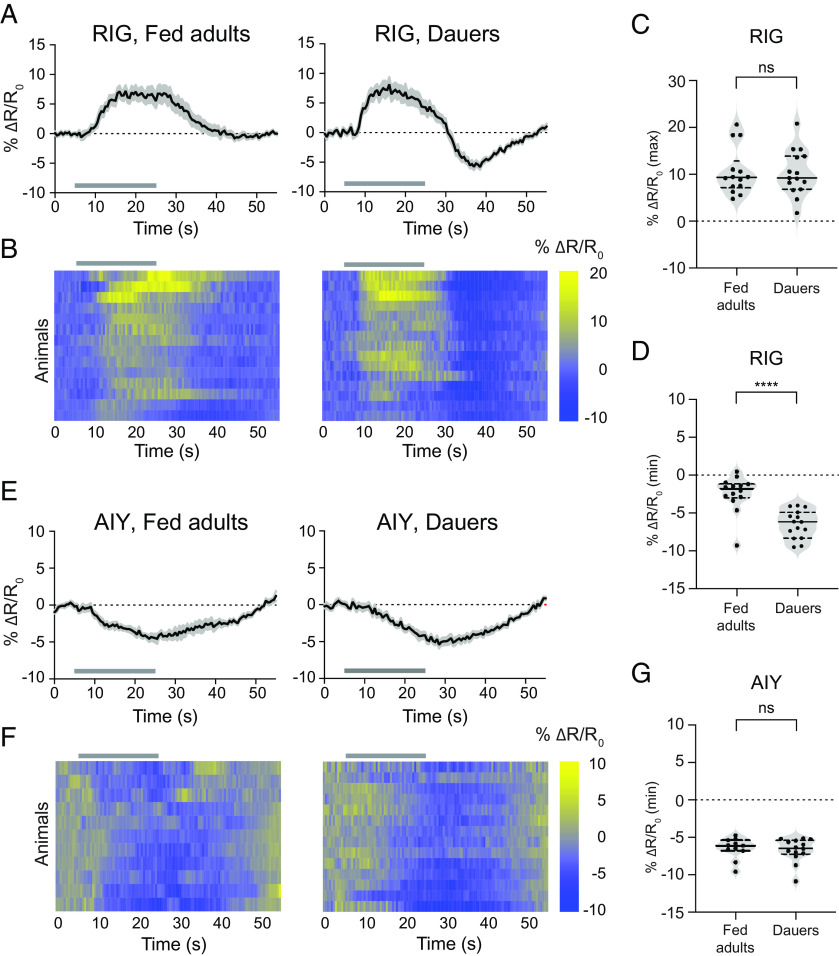
The RIG and AIY interneurons respond similarly to CO_2_ in well-fed adults and dauers. (*A*) CO_2_-evoked calcium responses of RIG in well-fed adults and dauers. Solid lines represent mean calcium responses; shading represents SEM. Gray bars indicate the timing and duration of the CO_2_ pulse. Calcium responses were measured using the ratiometric calcium indicator yellow cameleon YC3.60. Responses are to 15% CO_2_. (*B*) Heatmaps of RIG calcium responses. Each row represents the response of an individual animal. Gray bars indicate the timing and duration of the CO_2_ pulse. Response magnitudes are color-coded according to the scale (% ΔR/R_0_) shown to the right. Responses are ordered by hierarchical cluster analysis. (*C* and *D*) Quantification of the maximum (*C*) and minimum (*D*) responses of RIG in well-fed adults and dauers. ****P < 0.0001, Mann–Whitney test. ns = not significant (*P* = 0.8989), Welch’s *t* test. Each data point represents the response of a single animal. Solid lines in violin plots show medians and dotted lines show interquartile ranges. For *A*–*D*, n = 14 to 15 animals per life stage. (*E*) CO_2_-evoked calcium responses of AIY in well-fed adults and dauers. Conventions and conditions are as in panel *A*. (*F*) Heatmaps of AIY calcium responses. Conventions and conditions are as in panel *B*. (*G*) Quantification of the minimum responses of AIY in well-fed adults and dauers. ns = not significant (*P* = 0.7377), Mann–Whitney test. Conventions are as in panel *C*. For *E*–*G*, n = 11 to 14 animals per life stage.

We next investigated the CO_2_-evoked neural activity of four interneurons that are postsynaptic to BAG: RIG, AIY, AIB, and AVE (17–19). RIG was previously reported to undergo starvation-induced changes in CO_2_-evoked activity in adults––it displays excitatory CO_2_-evoked activity in well-fed adults but no activity in starved adults (27, 28). We monitored CO_2_-evoked calcium responses in dauers and found that the RIG neurons of dauers show excitatory responses that are similar to those of the RIG neurons of well-fed adults, even though well-fed adults and dauers show CO_2_ responses of opposite valence (Fig. 2 *A*–*C*). However, in dauers but not well-fed adults, the excitatory response in RIG was followed by an inhibitory response that initiated a few seconds after the termination of the CO_2_ pulse (Fig. 2 *A*, *B*, and *D*). As in well-fed adults (27), CO_2_-evoked responses in RIG were eliminated in dauers where the BAG neurons were genetically ablated (*SI Appendix*, Fig. S2 *A*–*C*), indicating that the CO_2_-evoked activity in dauer RIG neurons is dependent on sensory input from BAG. Thus, differences in chemosensory processing between starved adults and dauers are reflected at the level of RIG activity.

We then examined the CO_2_-evoked calcium responses of the AIY interneurons. Whereas AIY neurons in well-fed adults are inhibited by CO_2_, they show stochastic responses to CO_2_ in starved adults such that roughly equal proportions of animals display excitatory and inhibitory activities in AIY (27, 28). We found that the AIY neurons of dauers show inhibitory calcium responses to CO_2_ that are indistinguishable from those of well-fed adults (Fig. 2 *E*–*G*). To confirm that the inhibitory activity displayed by AIY in dauers was evoked by CO_2_, we measured AIY activity in response to an air control, where the CO_2_ pulse was replaced with an air pulse of equivalent duration. We found that the responses of AIY in both fed adults and dauers to CO_2_ were significantly different from the responses to the air control, confirming that the inhibitory responses were evoked by CO_2_ (*SI Appendix*, Fig. S2 *D*–*F*). Thus, like RIG, AIY shows distinct CO_2_-evoked calcium activity in starved adults vs. dauers.

**Fig. 3.**
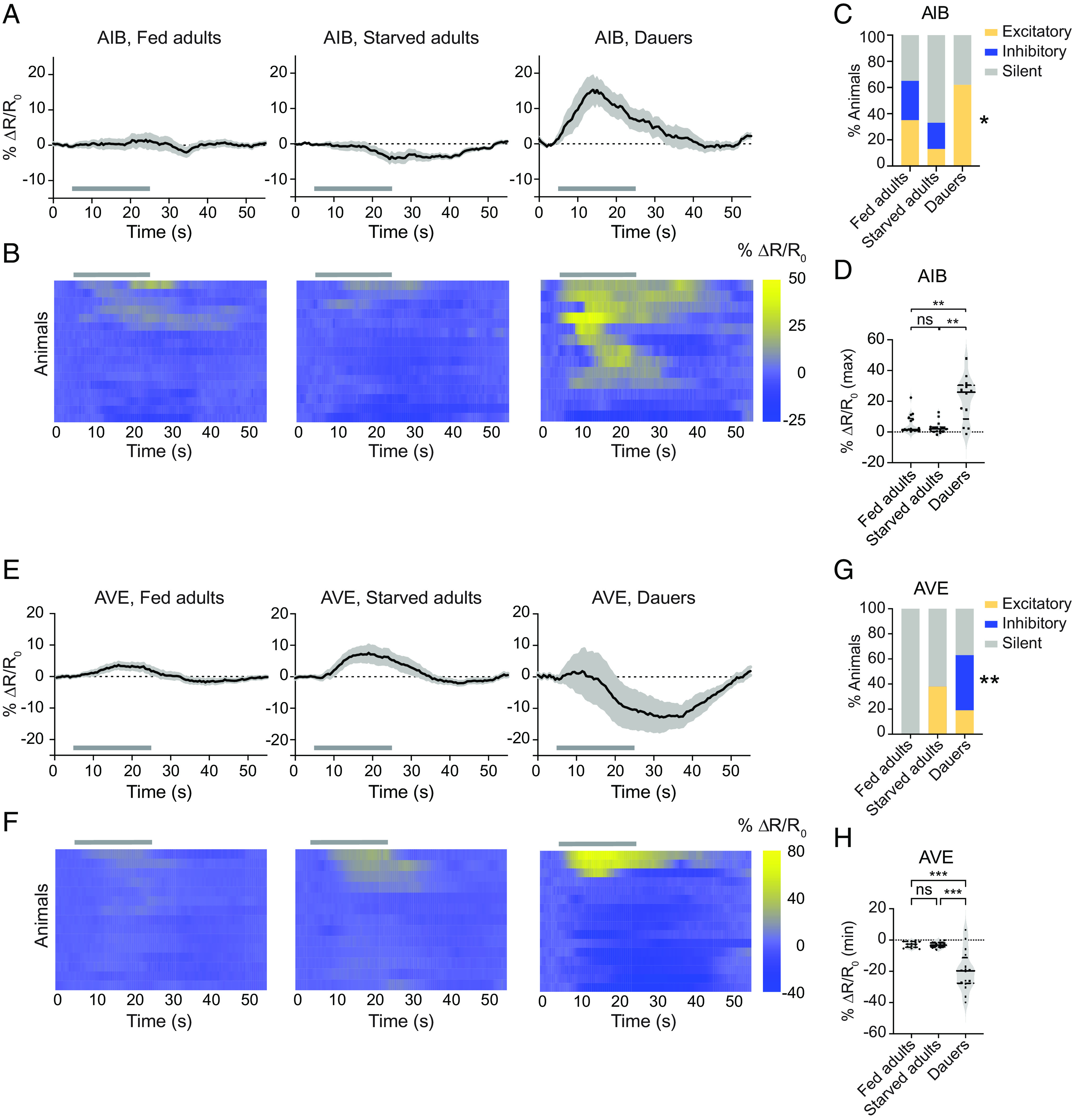
The AIB and AVE interneurons show distinct CO_2_-evoked calcium responses in dauers. (*A*) The AIB interneurons show robust excitatory activity in dauers. CO_2_-evoked calcium responses of AIB in well-fed adults, starved adults, and dauers. Solid lines represent mean calcium responses; shading represents SEM. Gray bars indicate the timing and duration of the CO_2_ pulse. Calcium responses were measured using the ratiometric calcium indicator yellow cameleon YC3.60. Responses are to 15% CO_2_. (*B*) Heatmaps of AIB calcium responses. Each row represents the response of an individual animal. Response magnitudes in the heatmaps are color-coded according to the scale (% ΔR/R_0_) shown to the right. Responses are ordered by hierarchical cluster analysis. Gray bars indicate timing and duration of the CO_2_ pulse. Calcium responses were measured using the ratiometric calcium indicator yellow cameleon YC3.60. Responses are to 15% CO_2_. (*C*) Categorical plot displaying the percentage of excitatory, inhibitory, and silent responses in AIB across life stages and conditions. Responses were categorized as either excitatory or inhibitory if the absolute value of the response exceeded 3 SDs of the response to an air control (28). *P < 0.05 for excitatory responses of dauers vs. starved adults, Fisher’s exact test. (*D*) Quantification of the maximum responses of AIB in well-fed adults, starved adults, and dauers. **P < 0.01, ns = not significant (*P *> 0.9999), Kruskal–Wallis test with Dunn’s posttest. For *A*–*D*, n = 13 to 17 animals per life stage and condition. (*E*) The AVE interneurons are primarily inhibited by CO_2_ in dauers. Calcium responses in the AVE neurons of well-fed adults, starved adults, and dauers. Conventions and conditions are as in panel *A*. (*F*) Heatmaps of AVE calcium responses. Conventions and conditions are as in panel *B*. (*G*) Categorical plot displaying the percentage of excitatory, inhibitory, and silent responses in AVE across life stages and conditions. Responses were categorized as in panel *C*. **P < 0.01 for inhibitory responses of dauers vs. starved adults, Fisher’s exact test. (*H*) Quantification of the minimum responses of AVE in well-fed adults, starved adults, and dauers. ***P < 0.001, ns = not significant (*P* = 0.9813), one-way ANOVA with Dunnett’s posttest. For *E*–*H*, n = 13 to 16 animals per life stage and condition. For *D* and *H*, each data point represents the response of a single animal. Solid lines in violin plots show medians and dotted lines show interquartile ranges.

In the case of AIB, we found that the interneuron shows small, infrequent responses to CO_2_ in both well-fed and starved adults (Fig. 3 *A*–*C*). In contrast, the AIB interneurons of dauers show robust excitatory calcium responses to CO_2_; these responses were observed in over 60% of the imaged animals (Fig. 3 *A*–*C*). The maximum peak amplitudes of these excitatory responses were significantly higher than those observed in adults (Fig. 3*D*). In addition, the AIB responses of dauers to CO_2_ vs. air were significantly different (*SI Appendix*, Fig. S3 *A*–*C*), confirming that the excitatory calcium responses of AIB in dauers are evoked by CO_2_. Finally, we found that the AVE interneurons are predominantly inhibited by CO_2_ in dauers; roughly 45% of dauers tested showed inhibitory responses in AVE (Fig. 3 *E*–*H*). None of the adults tested showed inhibitory responses, suggesting that AVE is inhibited specifically in dauers (Fig. 3*G*). Moreover, the minimum peak amplitudes of the CO_2_-evoked calcium responses were significantly lower in dauers relative to adults (Fig. 3*H*). The AVE responses we observed in dauers were significantly different between CO_2_ and air controls (*SI Appendix*, Fig. S4), indicating that they were evoked by CO_2_. Thus, the AIB and AVE interneurons appear to participate more reliably in the CO_2_ microcircuit of dauers than adults. Together, our findings demonstrate functional divergence in CO_2_-processing mechanisms at the interneuron level between starved adults and dauers.

### The Dauer-Specific Responses of AIB Require Gap Junctions and Insulin Signaling.

**Fig. 4.**
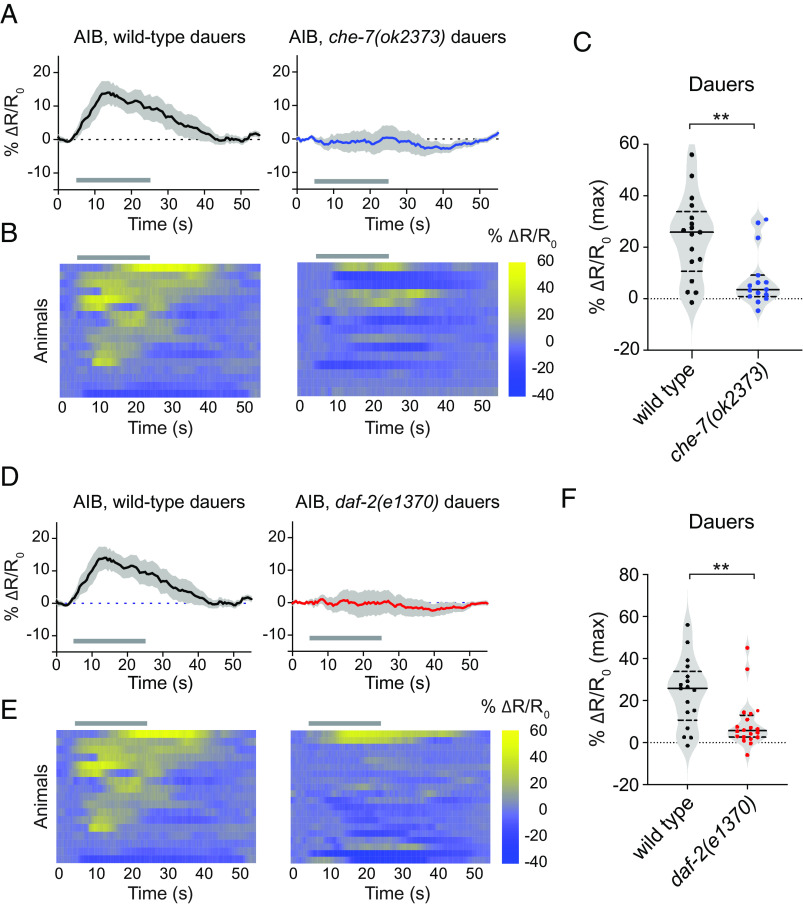
CO_2_-evoked activity in AIB is dependent on a BAG–AIB gap junction complex and insulin signaling. (*A*) Excitatory CO_2_-evoked calcium responses in AIB are largely eliminated in *che-7(ok2373)* mutant dauers. Calcium responses of the AIB neurons in wild-type dauers and *che-7(ok2373)* mutant dauers to CO_2_. Solid lines indicate mean calcium responses, and shading indicates SEM. Gray bars indicate timing and duration of the CO_2_ pulse. Calcium responses were measured using the ratiometric calcium indicator yellow cameleon YC3.60. Responses are to 15% CO_2_. (*B*) Heatmaps of AIB calcium responses. Each row represents the response of an individual animal. Gray bars indicate the timing and duration of the CO_2_ pulse. Response magnitudes in the heatmaps are color-coded according to the scale (% ΔR/R_0_) shown to the right. Responses are ordered by hierarchical cluster analysis. (*C*) Quantification of the maximum responses of AIB in wild-type and *che-7(ok2373)* dauers to CO_2_. Each data point represents the response of a single animal. Solid lines in violin plots show medians and dotted lines show interquartile ranges. **P < 0.01, Welch’s *t* test. n = 15 to 17 animals per genotype. (*D*) CO_2_-evoked calcium responses of the AIB neurons of wild-type and *daf-2(e1370)* mutant dauers. Conditions and conventions are as in panel *A*. (*E*) Heatmaps of AIB calcium responses. Conditions and conventions are as in panel *B*. (*F*) Quantification of the maximum responses of AIB in wild-type and *daf-2(e1370)* mutant dauers. **P < 0.01, Mann–Whitney test. n = 17 to 20 animals per genotype. Conventions are as in panel *C*.

What are the molecular mechanisms that contribute to the dauer-specific response properties of the CO_2_ microcircuit? To address this question, we focused on the AIB neurons, which respond robustly and consistently to CO_2_ in dauers but infrequently in starved adults (Fig. 3). A previous study found that AIB and BAG form gap junctions consisting of the subunits CHE-7 and INX-6 specifically in dauers (34). To test whether the excitatory CO_2_-evoked calcium responses in AIB in dauers arise due to dauer-specific gap junctions, we monitored CO_2_-evoked calcium activity in AIB in *che-7* mutant dauers. We found that the strong excitatory calcium responses were almost entirely absent in *che-7* dauers (Fig. 4 *A*–*C*), suggesting that the CO_2_-evoked excitatory response in dauers is dependent on the BAG–AIB electrical synapse.

We next sought to identify additional mechanisms that contribute to the dauer-specific response properties of the CO_2_ microcircuit. The insulin pathway plays an important role in regulating the developmental decision to enter the dauer state, and the altered physiology of dauers has been associated with changes in insulin signaling (30). We therefore tested whether insulin signaling also regulates the CO_2_ microcircuit of dauers. We found that the excitatory CO_2_-evoked calcium responses of AIB in wild-type dauers were largely eliminated in dauers lacking *daf-2*, which encodes the sole *C. elegans* homolog of the mammalian insulin/IGF receptor (30) (Fig. 4 *D*–*F*). Thus, AIB activity in dauers is dependent on insulin signaling. To determine whether the absence of CO_2_-evoked AIB activity in *daf-2* mutant dauers is due to a general physiological effect of the loss of insulin signaling, we monitored CO_2_-evoked activity in dauer RIG neurons. We found that the RIG neurons of *daf-2* mutant dauers showed normal CO_2_-evoked excitatory activity (*SI Appendix*, Fig. S5 *A*–*C*), indicating that *daf-2* specifically regulates the CO_2_-evoked activity of AIB. Since we did not observe detectable *daf-2* expression in AIB in dauers (*SI Appendix*, Fig. S6), *daf-2* likely functions cell-non-autonomously to modulate AIB activity. Moreover, since BAG–AIB gap junctions are present in *daf-2* dauers (34), *daf-2* does not modulate AIB activity through regulation of the BAG–AIB gap junction. Together, our results indicate that both changes in the electrical connectome and insulin signaling shape CO_2_ processing in dauers.

### Distinct Motor Programs Are Evoked by CO_2_ in Starved Adults and Dauers.

**Fig. 5.**
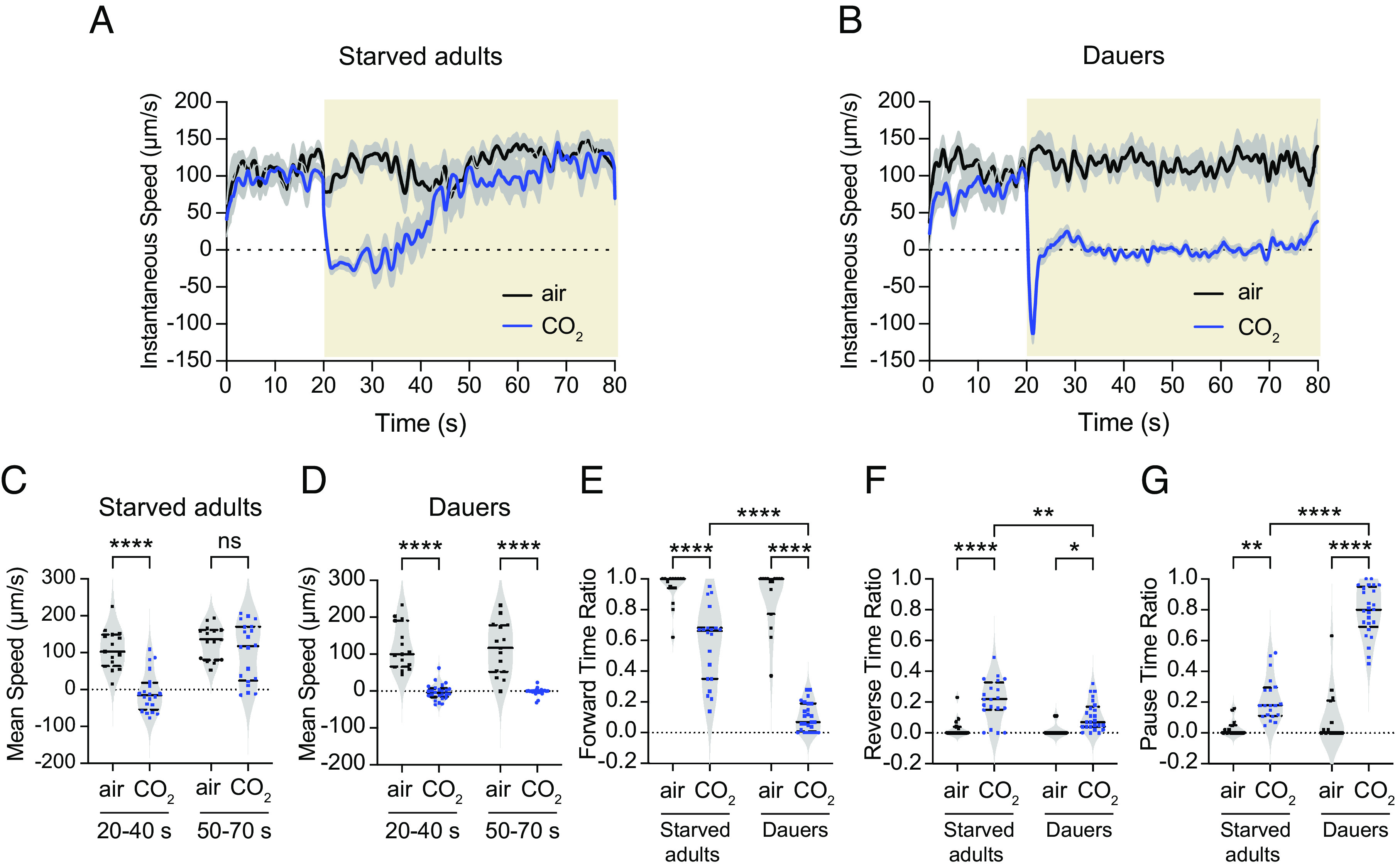
Distinct motor outputs are evoked by CO_2_ in starved adults vs. dauers (*A* and *B*) Changes in instantaneous speed of movement in starved adults (*A*) and dauers (*B*) in response to CO_2_. Black lines represent mean instantaneous speeds of animals exposed to a 20-s pulse of air (21% O_2_, balance N_2_) followed by a 60-s pulse of air, whereas blue lines represent mean instantaneous speeds of animals exposed to a 20-s pulse of air followed by a 60-s pulse of CO_2_ (2.5% CO_2_, 21% O_2_, balance N_2_). Shadings represent SEM. Yellow shaded boxes represent the timing and duration of the CO_2_ or air pulse. (*C* and *D*) Starved adults show a decrease in their mean speed during the first 20 s of CO_2_ exposure (*C*), whereas dauers show a decrease in their mean speed for the duration of CO_2_ exposure (*D*). (*E*) Both starved adults and dauers show reduced forward movement in response to CO_2_, but this effect is more pronounced for dauers. (*F*) Both starved adults and dauers show an increase in backward movement in response to CO_2_, but this effect is more pronounced for starved adults. (*G*) Both starved adults and dauers show an increased pause duration in response to CO_2_, but this effect is more pronounced in dauers. For *A*–*G*, n = 15 to 19 animals per life stage and condition. For *C*–*G*, each data point represents the response of a single animal. Solid lines in violin plots show medians and dotted lines show interquartile ranges. ****P < 0.0001, **P < 0.01, *P < 0.05, ns = not significant (*P *= 0.8028), two-way ANOVA with Sidak’s posttest.

Do the distinct functional properties of interneurons in dauers vs. starved adults affect CO_2_-evoked motor output at the two life stages? To address this question, we exposed animals to an acute CO_2_ pulse and video-recorded their movement (*SI Appendix*, Fig. S7 *A* and *B*) (35). We then tracked movement trajectories and quantified movement parameters (*SI Appendix*, Fig. S7 *C* and *D*). We found that starved adults exposed to CO_2_ reduced their speed for the first ~20 to 25 s of the CO_2_ pulse, after which they resumed movement at their prestimulus speed (Fig. 5*A*). This decline in speed was specific to CO_2_ since it did not occur when animals were exposed to an air pulse of equivalent duration (Fig. 5*A*). When dauers were exposed to the same CO_2_ pulse, they drastically reduced their speed for almost the entire duration of the CO_2_ pulse (Fig. 5*B*). This sharp decline in speed was also evoked by CO_2_ since it did not occur in response to an air pulse (Fig. 5*B*). The effect of CO_2_ on speed reduction was reversible since dauers resumed movement upon termination of the CO_2_ pulse (*SI Appendix*, Fig. S7*C*). We then compared the mean speeds of starved adults and dauers during the first 20 s following the start of the CO_2_ pulse, and during a later 20-s time window starting 30 s after the onset of the CO_2_ pulse. We found that the mean speed of starved adults was significantly reduced during the first 20 s of the CO_2_ pulse but not the later 20-s time window (Fig. 5*C*). In contrast, the mean speed of dauers was reduced during both time windows (Fig. 5*D*). In addition, whereas starved adults traveled a similar straight-line distance in response to CO_2_ vs. air, dauers traveled significantly less distance when exposed to CO_2_ (*SI Appendix*, Fig. S7*D*). Thus, CO_2_ exposure stimulates a more prolonged decrease in speed in dauers than starved adults.

We also quantified CO_2_-evoked changes in the directionality of movement for the two life stages. Both starved adults and dauers showed a significant reduction in the duration of forward movement in response to CO_2_ (Fig. 5*E*). However, forward movement duration was more strongly reduced in dauers than starved adults (Fig. 5*E*). Reverse movement duration increased in response to CO_2_ for both life stages, although the increase was less pronounced in dauers than starved adults (Fig. 5*F*). In addition, whereas CO_2_ stimulated an increase in pause time for both life stages, dauers paused for significantly longer than adults (Fig. 5*G*). Together, these results indicate that CO_2_ evokes distinct motor outputs in dauers vs. starved adults and is consistent with distinct CO_2_-evoked neural activity patterns across the two life stages.

### AIB Differentially Regulates CO_2_-Evoked Motor Output in Starved Adults vs. Dauers.

**Fig. 6.**
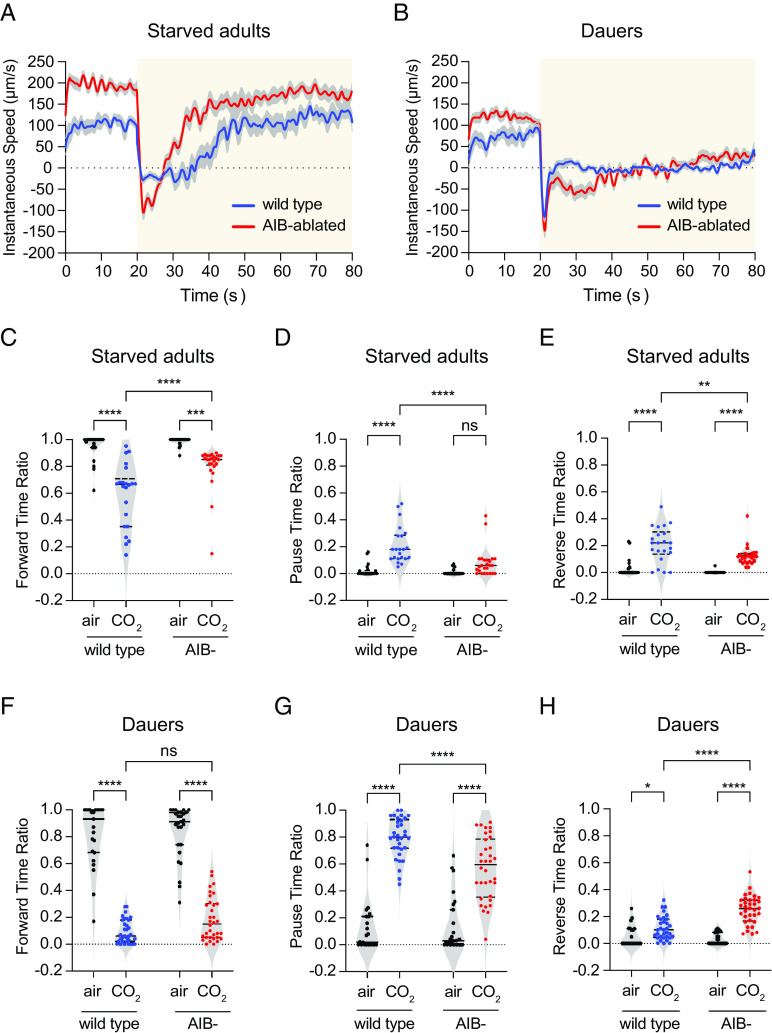
The AIB interneurons play distinct roles in regulating CO_2_-evoked motor output in starved adults vs. dauers. (*A* and *B*) Changes in instantaneous speed of (*A*) wild-type vs. AIB-ablated (AIB-) starved adults, and (*B*) wild-type vs AIB-ablated dauers in response to CO_2_. Yellow shaded boxes represent the timing and duration of the CO_2_ pulse. Blue and red lines represent mean instantaneous speeds of wild-type and AIB-ablated animals, respectively. Shadings represent SEM. Animals were exposed to 20 s pulses of air (21% O_2_, balance N_2_) followed by 60 s pulses of CO_2_ (2.5% CO_2_, 21% O_2_, balance N_2_). (*C*) Both wild-type and AIB-ablated starved adults show a reduction in forward movement duration in response to CO_2_, but this effect is less pronounced in AIB-ablated starved adults. (*D*) Wild-type starved adults but not AIB-ablated starved adults increase their pause duration in response to CO_2_. (*E*) Both wild-type and AIB-ablated starved adults show an increase in reverse movement duration in response to CO_2_, but this effect is less pronounced in AIB-ablated starved adults. (*F*) Both wild-type and AIB-ablated dauers show a similar reduction in forward movement duration in response to CO_2_. (*G*) Both wild-type and AIB-ablated dauers show an increase in pause duration in response to CO_2_, but this effect is less pronounced in AIB-ablated dauers. (*H*) Both wild-type and AIB-ablated dauers show an increase in reverse movement duration in response to CO_2_, but this effect is more pronounced in AIB-ablated dauers. For *A*–*H*, n = 21 to 34 animals per genotype, life stage, and condition. For *C*–*H*, each data point indicates the behavioral response of a single animal. Solid lines in violin plots show medians and dotted lines show interquartile ranges. ****P < 0.0001, **P < 0.01, *P < 0.05, ns = not significant (*P *> 0.07), two-way ANOVA with Sidak’s posttest.

We next investigated the role of AIB in driving CO_2_-evoked motor output in dauers vs. starved adults. We first confirmed that the promoter (*npr-9*) used to genetically target AIB showed the same expression pattern in starved adults and dauers (*SI Appendix*, Fig. S8). We then compared the CO_2_-evoked motor outputs of wild-type animals vs. animals where the AIB neurons are genetically ablated (35). We found that like wild-type starved adults, AIB-ablated starved adults terminated forward movement immediately after CO_2_ exposure (Fig. 6*A*). However, AIB-ablated starved adults reinitiated forward movement more rapidly than wild-type starved adults (Fig. 6*A*). In contrast, AIB-ablated dauers reversed during the first 5 s of CO_2_ exposure like wild-type dauers, but then exhibited more prolonged reversals than wild-type dauers (Fig. 6*B*). Thus, AIB ablation has distinct effects on the CO_2_-evoked locomotory patterns of starved adults vs. dauers.

To further investigate the role of AIB in driving CO_2_-evoked motor output in starved adults and dauers, we quantified the directionality of movement in wild-type vs. AIB-ablated starved adults and dauers. We found that for starved adults and dauers, both wild-type and AIB-ablated animals showed a decrease in forward time ratio and an increase in reverse time ratio in response to acute CO_2_ exposure relative to the air control. However, pause time ratio increased in AIB-ablated dauers but not AIB-ablated starved adults (Fig. 6 *C*–*H*). Interestingly, AIB-ablated starved adults reversed less than wild-type starved adults in response to CO_2_, whereas AIB-ablated dauers reversed more than wild-type dauers (Fig. 6 *E* and *H*). We further characterized this effect by quantifying the distance traveled in reverse in response to CO_2_ vs. an air control. For both starved adults and dauers, wild-type and AIB-ablated animals traveled more distance in reverse in response to CO_2_ than air (*SI Appendix*, Fig. S9). However, AIB-ablated starved adults traveled a shorter distance in reverse in response to CO_2_ than wild-type starved adults, whereas AIB-ablated dauers traveled a greater distance in reverse in response to CO_2_ than wild-type dauers (*SI Appendix*, Fig. S9). For both life stages, wild-type and AIB-abated animals traveled similar distances in reverse in response to the air controls, indicating that the effect of AIB on reversals is specific to CO_2_ (*SI Appendix*, Fig. S9). Thus, AIB exerts opposite effects on CO_2_-evoked reversals in starved adults vs. dauers: It promotes reversals in starved adults and suppresses reversals in dauers. In addition, we found that the CO_2_-evoked movement of *daf-2(e1370)* mutant dauers (where AIB excitatory activity is largely eliminated; Fig. 4 *D*–*F*) closely resembled that of AIB-ablated dauers (Fig. 6 *F*–*H* and *SI Appendix*, Fig. S10), suggesting that the CO_2_-evoked movement patterns of dauers arise at least in part due to the effects of insulin signaling on AIB activity. Together, our findings demonstrate a life stage–dependent change in the function of an interneuron in regulating chemosensory behavior.

Finally, we asked whether the dauer-specific excitatory response of AIB to CO_2_ is sufficient to account for the difference in CO_2_-evoked motor output between starved adults and dauers. We examined starved adults that expressed the bacterially derived voltage-gated sodium channel NaChBac specifically in AIB (AIB::NaChBac), leading to increased AIB excitability (36). We found that AIB::NaChBac starved adults showed a decrease in CO_2_-evoked forward movement and an increase in CO_2_-evoked reverse movement relative to wild-type starved adults (*SI Appendix*, Fig. S11); these responses were opposite to those of AIB-ablated starved adults (Fig. 6 *C* and *E*). However, unlike wild-type dauers, AIB::NaChBac starved adults did not show a dramatic increase in pause time in response to CO_2_ (Fig. 6*G* and *SI Appendix*, Fig. S11*B*). These results suggest that changes in AIB excitability alone in starved adults are not sufficient to generate dauer-like CO_2_-evoked motor output; rather, differences in motor output between the two life stages likely arise from the combined effects of multiple circuit components.

## Discussion

**Fig. 7.**
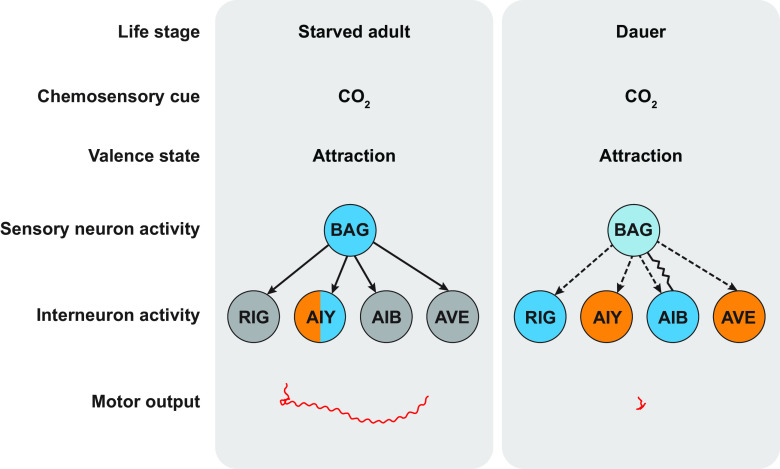
Functionally distinct microcircuits are involved in processing CO_2_ in starved adults vs. dauers. Differences in chemosensory processing are observed at the level of i) sensory neuron activity (the BAG neurons show reduced CO_2_-evoked activity in dauers), ii) interneuron activity (the same interneurons show distinct patterns of CO_2_-evoked activity in starved adults vs. dauers), and iii) motor output (distinct movement patterns are evoked by CO_2_ in starved adults vs. dauers). Excitatory and inhibitory neuronal activities are indicated by blue and orange shadings, respectively; nonresponsive neurons are indicated by gray shading. For BAG, the lighter shade of blue in dauers indicates reduced excitatory activity. For AIB and AVE, color codes indicate the predominant calcium response at the indicated life stages. The blue and orange shadings for AIY in starved adults indicate that the AIY neurons of starved adults respond stochastically to CO_2_ such that excitatory and inhibitory responses are generated at roughly equal frequencies (28). Solid arrows indicate chemical synapses in adults. Dashed arrows indicate putative chemical synapses in dauers; synapses are putative due to a lack of knowledge about the dauer connectome. The BAG–AIB gap junction in dauers is also shown. Red lines indicate the CO_2_-evoked movement trajectory of a single representative animal.

We have shown that the same chemosensory cue (CO_2_) is processed differently at two life stages that show the same valence state (CO_2_ attraction) (Fig. 7). Although CO_2_ is detected by the BAG sensory neurons in both starved adults and dauers, the functional architecture of the CO_2_ microcircuit differs at the sensory and interneuron levels. The BAG neurons in dauers show reduced excitatory calcium responses to CO_2_ relative to starved adults, and the interneurons downstream of BAG show distinct CO_2_-evoked calcium dynamics at the two life stages. Our findings demonstrate that functionally distinct microcircuits may underlie the detection and processing of a chemosensory cue across life stages sharing the same valence state, highlighting the need to consider context when dissecting chemosensory circuit function. Although it is unclear why dauers utilize a distinct CO_2_ microcircuit compared to starved adults, one possibility is that the dauer circuit reflects the need for dauers to display other dauer-specific behaviors, such as nictation and dauer recovery (30). It remains to be determined whether CO_2_ acts as a sensory cue to drive these or other dauer-specific behaviors.

The BAG neurons show reduced CO_2_-evoked activity in dauers compared to starved adults that is independent of *gcy-9* expression (Fig. 1 *B*–*D* and *SI Appendix*, Fig. S1 *A*–*B*). Previous studies have shown that CO_2_-evoked BAG activity in adults may be suppressed by molecular mechanisms that operate downstream of GCY-9 or inhibitory signaling from downstream interneurons (37, 38). Whether similar mechanisms contribute to the reduced BAG response of dauers or whether other sensory cues are required to reach maximal activity levels remains to be elucidated. It is also possible that the reduced BAG activity of dauers is the result of reduced entry of CO_2_ through the thicker cuticle of dauers.

At the interneuron level, we have shown that the dauer-specific response properties of the AIB interneurons require a dauer-specific BAG–AIB electrical synapse (Fig. 4 *A*–*C*). The BAG neurons form a chemical synapse with AIB in adults (17), but the presence of this synapse does not appear to be sufficient for robust CO_2_-evoked activity in AIB (Fig. 3 *A*–*D*). It is possible that the electrical synapses between BAG and AIB in dauers lead to alterations in the composition and/or function of the chemical synapses between BAG and AIB, as has been shown for a different synapse in *C. elegans* (39). The insulin receptor DAF-2 appears to act non-cell-autonomously to modulate AIB activity in dauers (Fig. 4 *D*–*F* and *SI Appendix*, Fig. S6). In future studies, it will be interesting to determine whether DAF-2 has distinct effects on CO_2_ attraction in starved adults vs. dauers. Moreover, identifying the site of action of DAF-2, as well as the signaling pathways that act downstream of DAF-2 to functionally modulate the CO_2_ microcircuit in dauers, would provide additional insight into how insulin signaling sculpts chemosensory behaviors.

While our results illustrate that the interneurons downstream of BAG show distinct CO_2_-evoked activity patterns in dauers vs. adults, the precise roles of each of these interneurons in driving CO_2_-evoked behavior remain to be determined. In adults, the AIY interneurons promote forward movement (40, 41). Thus, the stochastic excitatory activity of AIY in starved adults (28) may cause starved adults to resume forward movement after reversing or pausing upon initial CO_2_ exposure. The inhibitory activity of AIY in dauers (Fig. 2 *E*–*G*) may suppress forward movement and thereby promote CO_2_-evoked pausing (Fig. 5*B*). In the case of RIG, its role in regulating movement at any life stage is poorly understood. The finding that well-fed adults and dauers show similar CO_2_-evoked excitatory activity in RIG despite showing CO_2_ responses of opposite valence (Fig. 2 *A*–*C*) raises the possibility that RIG may play a different role in regulating movement at the two life stages. However, in dauers but not adults, the excitatory response in RIG was followed by an inhibitory response, raising the possibility that this inhibitory response promotes dauer-specific CO_2_-evoked behavior.

The AIB interneurons promote reversals in starved adults but suppress reversals in dauers upon acute CO_2_ exposure (Fig. 6 and *SI Appendix*, Fig. S11). Prior studies have demonstrated a role for AIB in promoting basal reversals in adults (42–45) as well as dauers (34). Thus, AIB appears to have a context-dependent role in regulating reversal behavior in dauers. Moreover, given the opposite roles of AIB in regulating CO_2_-evoked reversals in starved adults vs. dauers, it is possible that the CO_2_-evoked excitatory activity of AIB in dauers and the lack of CO_2_-evoked activity in AIB in starved adults both serve to suppress reversals upon acute CO_2_ exposure, thereby promoting CO_2_ attraction. In the case of AVE, excitatory activity in AVE is associated with reversals in adults (43, 46). Thus, it is possible that the silencing of AVE in starved adults and the inhibition of AVE in dauers both serve to suppress reversals in response to CO_2_. However, the functional consequence of CO_2_-evoked inhibition of AVE, as opposed to silencing, in dauers remains unclear.

Together, our results demonstrate that divergent CO_2_-evoked neural mechanisms operate at the sensory and interneuron levels in dauers vs. starved adults despite the two life stages sharing the same valence state. In future studies, it will be interesting to determine whether different mechanisms also underlie the same chemosensory valence state in other organisms, including humans. In addition, dauer larvae are developmentally similar to the infective larvae of parasitic nematodes (47), which infect over one billion people worldwide and cause some of the most devastating neglected tropical diseases (48, 49). The infective larvae of multiple parasitic nematode species use CO_2_ as a host-seeking cue (20, 24, 50–52), but the neural mechanisms that drive these responses remain unknown. Thus, a better understanding of how *C. elegans* responds to CO_2_ may lead to new strategies for controlling parasitic nematode infections.

## Materials and Methods

Behavioral assays were performed essentially as previously described, with some modifications (35). Calcium imaging was performed as previously described (27, 28). Statistical tests were performed using GraphPad Prism v9.3.1. All data from this study are available on GitHub (https://github.com/HallemLab/Banerjee_et_al_2023). For detailed information on all materials and methods, see *SI Appendix*, *Materials and Methods*.

## Supplementary Material

Appendix 01 (PDF)

Dataset S01 (XLSX)

## Data Availability

All study data are included in the article and/or supporting information or are available on GitHub (https://github.com/HallemLab/Banerjee_et_al_2023) (53).
