# Supplementary material for: Differential processing of a chemosensory cue across life stages sharing the same valence state in Caenorhabditis elegans
Source: Proc Natl Acad Sci U S A. 2023 May 1;120(19):e2218023120. doi: 10.1073/pnas.2218023120 (PMC10175840; doi:10.1073/pnas.2218023120)
Supplement: Supplementary file 1 — Appendix 01 (PDF) [file pnas.2218023120.sapp.pdf]

PNAS

Supporting Information for

Differential processing of a chemosensory cue across life stages sharing the same valence state in *Caenorhabditis elegans*

Navonil Banerjee, Pei-Yin Shih, Elisa J. Rojas Palato, Paul W. Sternberg, and Elissa A. Hallem

Elissa Hallem
Email: ehallem@ucla.edu

Paul Sternberg
Email: pws@caltech.edu

This PDF file includes:

Supporting text
Figures S1 to S11
Table S1
Legend for Dataset S1
SI References

Other supporting materials for this manuscript include the following:

Dataset S1

SI Materials and Methods

C. elegans strains

Worms were cultured and maintained on 2% Nematode Growth Media (NGM) plates seeded with *Escherichia coli* OP50 bacteria at ambient temperature (~22°C) and CO_2 (~0.038%) following standard procedures (1, 2). The temperature-sensitive, dauer-constitutive strains CB1370, EAH382, and EAH383 were maintained at 15ºC but were moved to ambient temperature (~22°C) at least 5 days prior to experiments to minimize any effects of temperature shifts on behavior or neuronal calcium responses. Strains used in this study are listed in Table S1. The strains EAH381 and EAH383 were generated by crossing EAH259 *Ex[*P*odr-2(1b)::YC3.60;* P*lin-44::GFP]* to RB1834 *che-7(ok2373)* and CB1370 *daf-2(e1370)*, respectively. The strain EAH382 was generated by crossing PS6028 *syEx1134[*P*twk-3::YC3.60;* P*pax-2::GFP]* to CB1370 *daf-2(e1370)*. The strain EAH409 was generated by crossing QQ202 *daf-2*(*cv20*[*daf-2*::*GFP*]) to OH13918 *otIs643*[P*npr-9::TagRFP*] (a gift from Oliver Hobert).

Preparation of animals for CO_2 chemotaxis assays

Well-fed adults: Assays were performed with young adults (~1 day old) as previously described (3, 4). Briefly, animals were collected in a 65 mm Syracuse watch glass by washing them off plates with M9 buffer. Animals were washed twice with M9 buffer and then once with ddH_2O in the watch glass. Animals were then gently transferred onto a small rectangular piece of Whatman filter paper, which was used to transport them to the center of a 10 cm 2% NGM plate without food for chemotaxis assays.

Starved adults: Young adults were washed in a watch glass as described above and then starved on a 10 cm 2% NGM plate without bacterial food for 3 h as previously described (4). Animals were placed within an annular ring of filter paper soaked in 20 mM copper chloride ($CuCl_2$) solution to keep them from crawling off the edges of plates, since copper is aversive to *C. elegans* (5). After 3 h of starvation, the $CuCl_2$ ring was removed; the animals were collected from the plate and washed twice in M9 and once in ddH_2O in a watch glass. Animals were then transferred onto the center of a 10 cm 2% NGM plate for chemotaxis assays using a piece of Whatman filter paper.

Dauers: To generate dauer larvae, 8-10 young adults were transferred to 2% NGM plates with a lawn of OP50 and left for 10-14 days at room temperature until the OP50 on the plates was depleted. Dauers were differentiated from other life stages based on their resistance to sodium dodecyl sulphate (SDS), as described previously (6). Briefly, animals were washed off food-depleted plates with dH_2O into a 15 mL conical tube and centrifuged at 1000 rpm for 60-90 s. The supernatant was discarded without disturbing the worm pellet. The tube was then filled with 5 mL 1% SDS solution and gently mixed on a rotator at room temperature for 15 min. Following SDS treatment, the tube was centrifuged at 1000 rpm for 60-90 s, and the SDS was removed without disturbing the pellet. 10 mL dH_2O was then added to the tube, which was mixed thoroughly and then centrifuged at 1000 rpm for 60-90 s. Washes with 10 mL dH_2O were repeated twice for a total of three washes. Finally, dauers were transferred in water drops onto 2% NGM plates for chemotaxis assays.

CO_2 chemotaxis assays

CO_2 chemotaxis assays were performed as previously described (3, 4). Animals were placed onto the center of a 10 cm 2% NGM plate at the start of assay. The CO_2 stimulus (the desired concentration of CO_2, 21% O_2, balance N_2) and air stimulus (21% O_2, balance N_2) were pumped through holes in opposite sides of the plate lid to establish a CO_2 gradient. Gas stimuli were delivered using a syringe pump (PHD 2000, Harvard Apparatus) through ¼-inch flexible PVC tubing at flow rates of 2 mL/min (for adult assays) or 0.5 mL/min (for dauer assays). Assays ran for 20 min (for adults) or 1 h (for dauers). At the end of each assay, the number of animals within a 20 mm diameter circle under each gas inlet (in the case of adults) or within 30 mm segments on both sides of the plate (in the case of dauers) were counted. A chemotaxis index (CI) was then calculated as:

$$CI = \frac{\text{\# animals in the } CO_2 \text{ region - \# animals in the air control region}}{\text{\# animals in the } CO_2 \text{ region + \# animals in the air control region}}$$

To control for directional bias due to room vibration or other sources, assays were conducted in pairs, with the CO_2 gradients in opposite orientations for the two plates. If the absolute value of the difference in CI between two assays in a pair was $\geq$0.9, both assays were discarded as behavior was assumed to be impacted by directional bias. Assays were also discarded if fewer than 7 animals moved to the combined scoring regions.

Calcium imaging

Calcium imaging experiments were performed as previously described (3, 4, 7) using the genetically encoded calcium indicator yellow cameleon YC3.60 (8). To starve animals prior to imaging, young adults expressing YC3.60 were picked from NGM plates with OP50 to NGM plates without bacteria and allowed to crawl for 1 min to remove residual bacteria. Animals were then transferred to 2% NGM plates (without food) lined with an annular ring of $CuCl_2$-soaked Whatman paper and starved for 3-6 h before imaging. Dauers for imaging were isolated by SDS treatment as described above.

For imaging, adults were immobilized onto a freshly made 2% agarose pad (made with 10 mM HEPES) on a cover glass using Meridian Surgi-Lock 2oc surgical glue. To immobilize dauers, which are resistant to glue, a 2% agarose pad (made with 10 mM HEPES) was made on a cover glass and left to dry overnight. A single dauer was transferred in a water droplet onto the dry agarose pad. The water droplet was then absorbed using a piece of Whatman paper, leaving the dauer behind. This resulted in attachment of the dauer to the dry agarose pad, which restricted its movement during imaging. A chamber fabricated from a 6 cm Petri dish with a 15 mm hole at the base and two 5 mm holes on diametrically opposite sides for gas inlets was placed around the animal and secured onto the cover glass with beeswax. The chamber was humidified using wet tissue wipes. Gases were delivered into the chamber at flow rates of 0.7-0.8 L/min (controlled by a flow meter) from two gas tanks fitted with valves controlled by a ValveBank TTL pulse generator. An air pulse (21% O_2, balance N_2) was delivered for 20 s, followed by a 20 s CO_2 pulse (15% CO_2, 21% O_2, balance N_2) and then another air pulse (21%

O_2, balance N_2) for 30 s. For air controls, the CO_2 pulse was replaced with an air pulse of equivalent duration (20 s). Imaging was performed using a Zeiss AxioObserver A1 inverted microscope equipped with a 40x EC Plan-NEOFLUAR lens and a Hamamatsu C9100 EM-CCD camera. Images were acquired in the YFP and CFP channels at 2 frames/s using Zeiss AxioVision software. The emission image was passed through a DV2 beam splitter (Photometrics) as previously described (3, 4).

Image analyses were performed using AxioVision software and Microsoft Excel. Images were analyzed by selecting regions of interest (ROIs) that consisted of either the soma (BAG, RIG, AVE) or process (AIY, AIB) of the neuron of interest and a background region. For AIY imaging, the synapse-rich part of the process previously designated as zone 2 (9) was selected as the ROI. For AIB imaging, a segment of the process where spatiotemporal expression of INX-6 was observed in dauers (10) was selected as the ROI. The average intensity for YFP and CFP of the background region was subtracted from the average intensity for YFP and CFP of the soma or process. YFP values were adjusted to correct for CFP signal bleed-through, and the YFP/CFP ratio was then calculated. The data were baseline-adjusted for linearity using the 10 s before and after the gas stimulus as the baseline. For each dataset, the different genotypes, life stages, or conditions were tested in parallel.

For quantification, the response period was defined as the time interval beginning with the onset of the CO_2 pulse and ending 10 s after the offset of the CO_2 pulse. The % $\Delta R/R_0$ (max) and % $\Delta R/R_0$ (min) values were calculated during the response period. For AVE imaging, categorization of responses as excitatory, inhibitory, or silent was performed using threshold values generated from air control experiments performed for each life stage or condition. Maximum and minimum threshold values were set as 3 standard deviations above the mean % $\Delta R/R_0$ (max) air response or 3 standard deviations below the mean % $\Delta R/R_0$ (min) air response, respectively. A CO_2 response with a % $\Delta R/R_0$ (max) value higher than the maximum threshold value was designated as excitatory; a response with a % $\Delta R/R_0$ (min) value lower than the minimum threshold value was designated as inhibitory. A CO_2 response where the most extreme % $\Delta R/R_0$ values were within the maximum and minimum thresholds was designated as silent. For all recordings except RIG, recordings were excluded from quantification if one or more of the % $\Delta R/R_0$ values during the 5 s interval preceding CO_2 onset was outside of the range defined by ±3 standard deviations from the mean % $\Delta R/R_0$ (max) or % $\Delta R/R_0$ (min) values of an air stimulus during the same time interval (air stimuli were delivered to separate sets of worms in control experiments). All excluded imaging traces are available on GitHub. Heatmaps were generated using GraphPad Prism v9.1.0. Responses within heatmaps were ordered by hierarchical clustering analysis using the web-based tool Heatmapper (11), using Euclidean distance as a similarity measure.

Microscopy
For starved adults, 1-day old adults (L4-synchronized) were starved for 3 h prior to imaging as described above. Dauers for imaging were isolated by SDS treatment as described above. Animals were anesthetized using 10 mM levamisole and mounted on 5% agarose pads on glass slides. Imaging was performed on a Zeiss AxioObserver inverted wide-field fluorescent

microscope equipped with a Colibri 7 for LED fluorescence illumination, a Plan-APOCHROMAT 20X or 40X objective lens, a Hamamatsu ORCA-Flash 4.0 camera and Zen software (Zeiss). For Fig. S8, images were captured as z-stacks using a 20X objective and maximal intensity projections were constructed using Fiji (12). For Fig. S1 and S6, images were captured using a 40X objective at a single focal plane and processed using Fiji (12). Fluorescence intensity measurements in Fig. S1B and S1D were performed using Fiji (12). For each image, the mean fluorescence intensity of a background region of interest was subtracted from the mean fluorescence intensity of the cell body, and the resulting intensity values were plotted using GraphPad Prism v9.3.1.

Acute CO_2 assays and behavioral tracking

One-day-old adults (age-synchronized at the L4 larval stage) were used for acute CO_2 assays. To starve animals prior to the assay, approximately 30 adults were picked from NGM plates with OP50 bacteria to unseeded plates and allowed to crawl for approximately 1 min to remove residual bacteria. Animals were then transferred to unseeded 2% NGM plates lined with an annular ring of $CuCl_2$-soaked Whatman paper and starved for 3 h prior to the start of the assay. Dauers were isolated by SDS treatment as described above and placed on unseeded 2% NGM plates at least 1 h prior to the start of the assay.

Assays were performed using 15 cm unseeded 2% NGM plates at room temperature. 8-10 animals were individually picked to assay plates and left to acclimate for approximately 5 min. Since movement in dauers is interrupted by extensive periods of spontaneous pausing, only dauers that were actively moving were picked to assay plates. A chamber with a 6 cm viewing arena was connected to gas tubing (Fig. S7A), then placed on top of the assay plate as previously described (13). Premixed gases were delivered into the chamber at a flow rate set to 25 mL/min (controlled by a VWR 97004-780 flow meter) from gas tanks (Airgas) fitted with valves controlled by a ValveBank. Prior to videorecording, air (21% O_2, balance N_2) was delivered into the chamber for 2 min to acclimate animals to gas flow. Video recording started immediately post-acclimation, when animals were exposed to a pulse train of air (21% O_2, balance N_2) for 20 s, followed by CO_2 (2.5% CO_2, 21% O_2, balance N_2) for 60 s and then air (21% O_2, balance N_2) for 20 s (Fig. S7B). For air controls, the CO_2 pulse was replaced with an air pulse of equivalent duration. Animals were videorecorded for a total of 100 s using a Leica S9D microscope (equipped with a 0.5x supplementary lens, a 300 mm M-series Focus drive and a TL3000 Ergo transmitted light base) with an attached Basler Ace 20-megapixel acA5472-17µm camera (mounted on a 0.63x Leica adapter lens for assays with adults, or a 1x Leica adapter lens for assays with dauers). Image sequences were captured at 10 frames/s using Pylon viewer software (Basler). Image sequences were processed to generate videos using Fiji (12).

Tracking and analyses of movement were performed using WormLab 2022.1.1 (MBF Bioscience LLC, Williston, VT USA). Videos were optimally thresholded to detect animals and their movement was automatically tracked. Movement parameters such as instantaneous speed, mean speed, and directionality (forward or reverse) were calculated from the tracks of individual animals. Instantaneous speed was calculated as a moving average speed over 3 s

that was smoothed using locally weighted polynomial regression. Forward or reverse movement was defined as movement in either direction at a minimum absolute speed of 30 μm/s that lasted for at least 1 s. Pause was defined as movement at absolute speeds below 30 μm/s for at least 1 s. Forward, reverse, or pause duration ratios were calculated as the time spent moving (in a particular direction) or pausing as a fraction of the total time over which the animal was tracked. Animals that could not be tracked for at least 57 s (out of 60 s) during the CO_2 (or air control) pulse were excluded from analysis. Since dauer movement is interrupted by prolonged bouts of spontaneous pausing, only dauers that were actively moving during the first 20 s air pulse were considered for tracking and analysis. Data were graphed using GraphPad Prism v9.3.1.

Statistical analysis
Statistical tests were performed using GraphPad Prism v9.3.1. Specific statistical tests used are indicated in the figure legends. Normality was determined using a D'Agostino-Pearson omnibus normality test; if data were not normally distributed, non-parametric tests were used. Power analyses were performed using G*Power v3.1.9.6 (14).

Data Availability
The data that support the findings of this study are available on GitHub (https://github.com/HallemLab/Banerjee_et_al_2023).

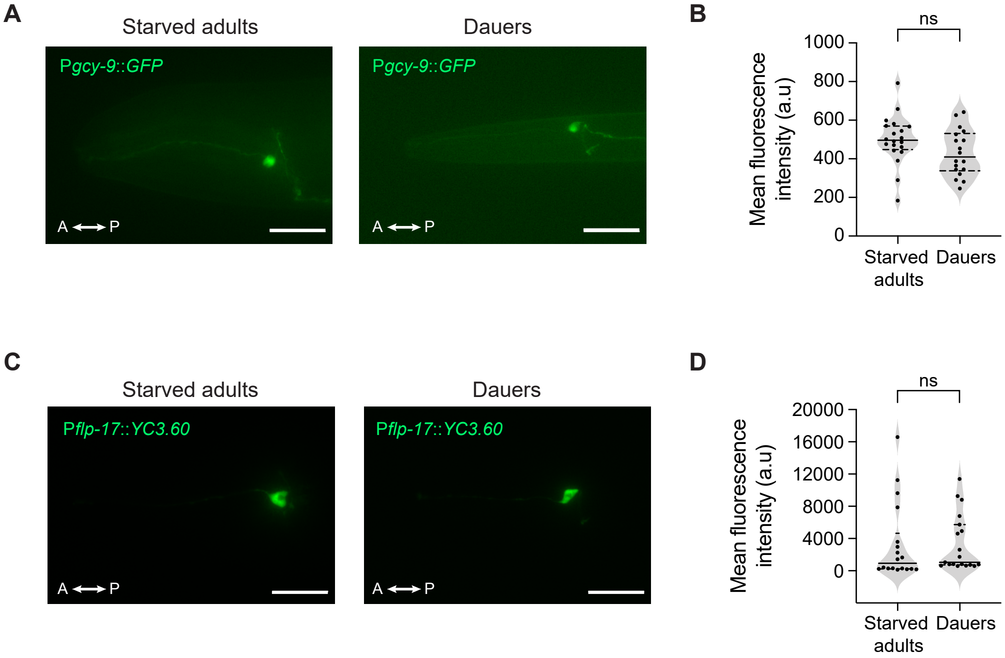

Fig. S1. The P*gcy-9::GFP* and P*flp-17::YC3.60* transgenes show similar expression levels in starved adults and dauers. (A) Epifluorescent images of the BAG neurons of starved adults and dauers expressing a P*gcy-9::GFP* transgene. Scale bar = 25 μm. **(B)** Quantification of mean GFP fluorescent intensities in the BAG cell bodies of starved adults vs. dauers. Each data point indicates the fluorescent intensity measurement of a single animal. Solid lines in violin plots show medians and dotted lines show interquartile ranges. ns = not significant (p = 0.0939), Welch's t-test. n = 18-20 animals per life stage. a.u. = arbitrary units. **(C)** Epifluorescent images of the BAG neurons of starved adults and dauers expressing a P*flp-17::YC3.60* transgene. Scale bar = 25 μm. **(D)** Quantification of mean fluorescent intensities of YC3.60 in the BAG cell bodies of starved adults vs. dauers. ns = not significant (p = 0.1785), Mann-Whitney test. n = 18-19 animals per life stage. Conventions are as in panel B.

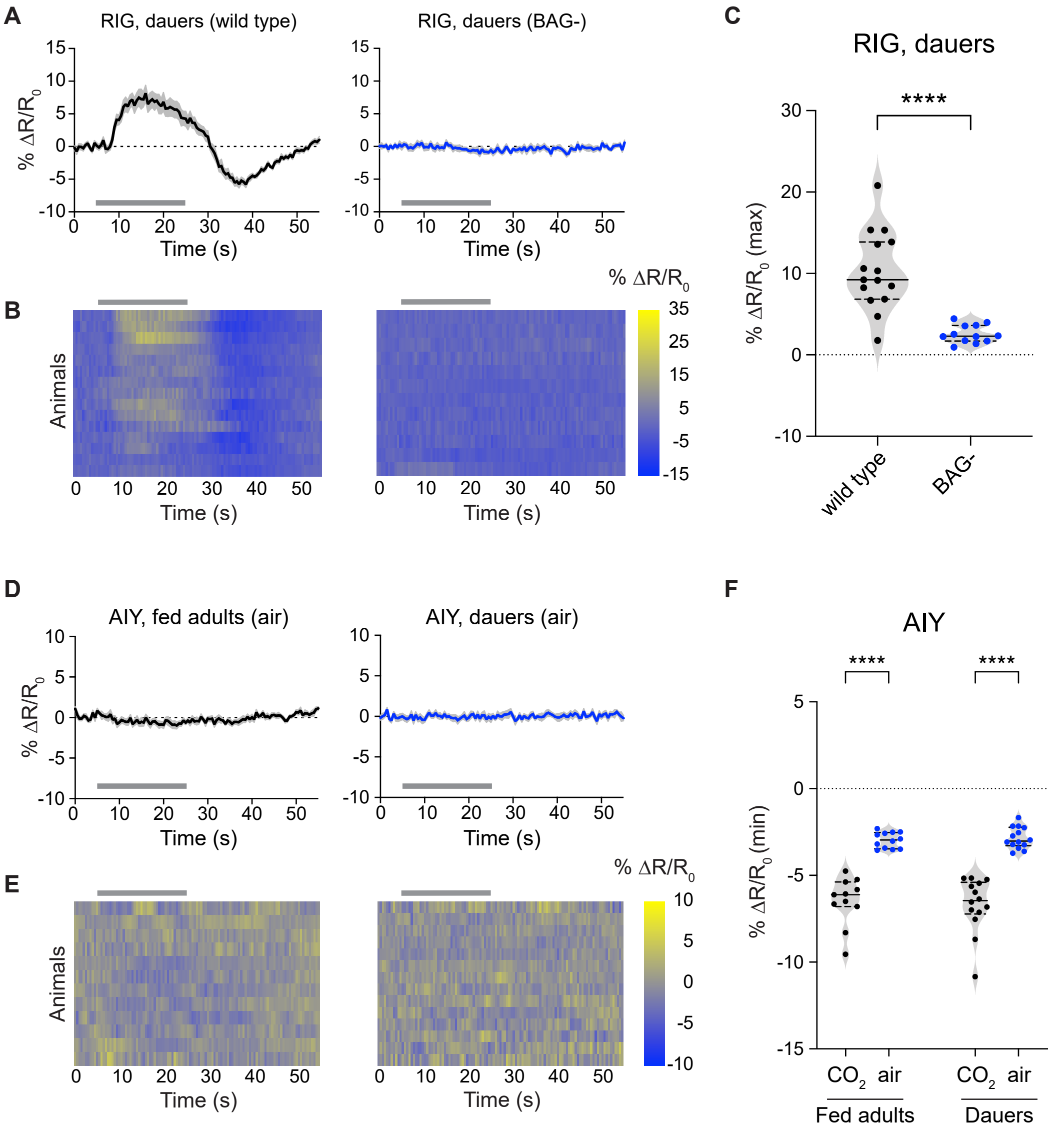

Fig. S2. RIG activity in dauers is dependent on BAG signaling. (A) Calcium responses of RIG neurons in wild-type vs BAG-ablated (BAG-) dauers. Solid lines indicate mean calcium responses and shading indicates SEM. Gray bars indicate timing and duration of the CO_2 pulse. Calcium responses were measured using the ratiometric calcium indicator yellow cameleon YC3.60. Responses are to 15% CO_2. **(B)** Heatmaps of RIG calcium responses. Each row indicates the response of an individual animal. Gray bars indicate the timing and duration of the CO_2 pulse. Response magnitudes in the heatmaps are color-coded according to the scale (% $\Delta R/R_0$) shown to the right. Responses are ordered by hierarchical cluster analysis. **(C)** Quantification of the maximum responses of RIG in wild-type and BAG- dauers. Each data point represents the response of a single animal. Solid lines in violin plots show medians and dotted lines show interquartile ranges. *****p*<0.0001, Welch's t-test. n = 12-15 animals per genotype. **(D)** Calcium responses of AIY neurons in fed adults and dauers in response to an air control. Responses are to 21% O_2. Other conditions and conventions are as in panel A. **(E)** Heatmaps of AIY calcium responses. Responses are to 21% O_2. Other conditions and conventions are as in panel B. **(F)** Quantification of the minimum responses of AIY in fed adults and dauers in response to CO_2 vs. air. *****p*<0.0001, 2-way ANOVA with Sidak's post-test. n = 11-14 animals per life stage and condition. Conventions are as in panel C.

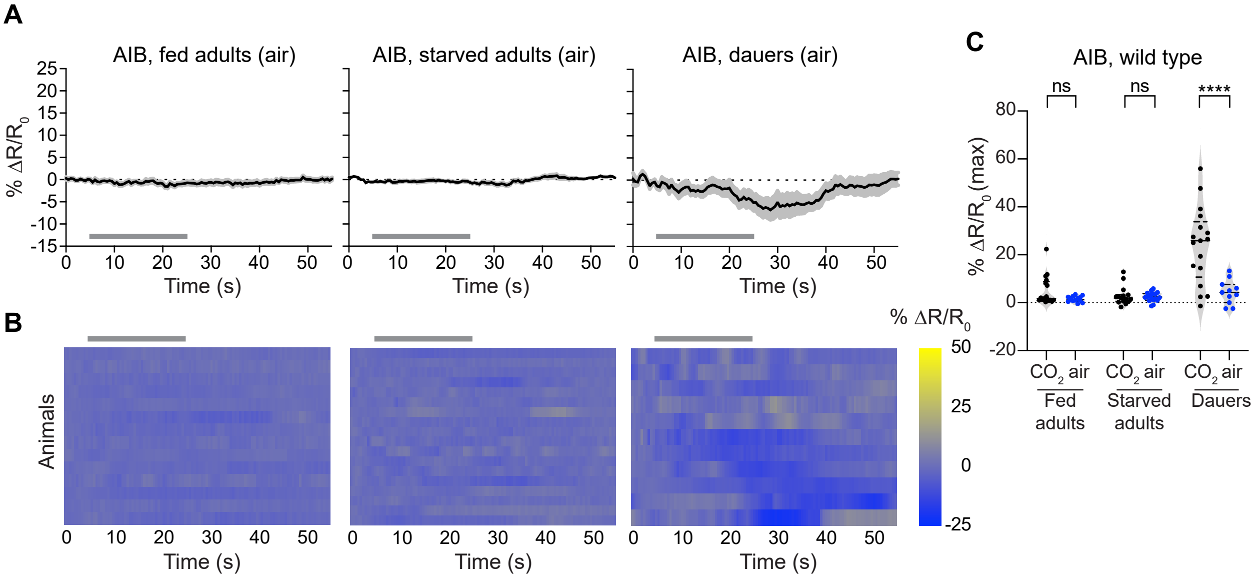

Fig. S3. AIB activity in dauers is evoked by CO_2. (A) Calcium responses of AIB neurons in well-fed adults, starved adults, and dauers in response to an air control. Solid lines indicate mean calcium responses and shading indicates SEM. Gray bars indicate timing and duration of the CO_2 pulse. Calcium responses were measured using the ratiometric calcium indicator yellow cameleon YC3.60. Responses are to 21% O_2. **(B)** Heatmaps of AIB calcium responses. Each row represents the response of an individual animal. Gray bars indicate the timing and duration of the CO_2 pulse. Response magnitudes in the heatmaps are color-coded according to the scale (% $\Delta R/R_0$) shown to the right. Responses are ordered by hierarchical cluster analysis. **(C)** Quantification of the maximum responses of AIB in well-fed adults, starved adults, and dauers to CO_2 vs. an air control. Each data point represents the response of a single animal. Solid lines in violin plots show medians and dotted lines show interquartile ranges. *****p*<0.0001, two-way ANOVA with Sidak's post-test. ns = not significant (*p*>0.5). n = 11-18 animals per life stage and condition.

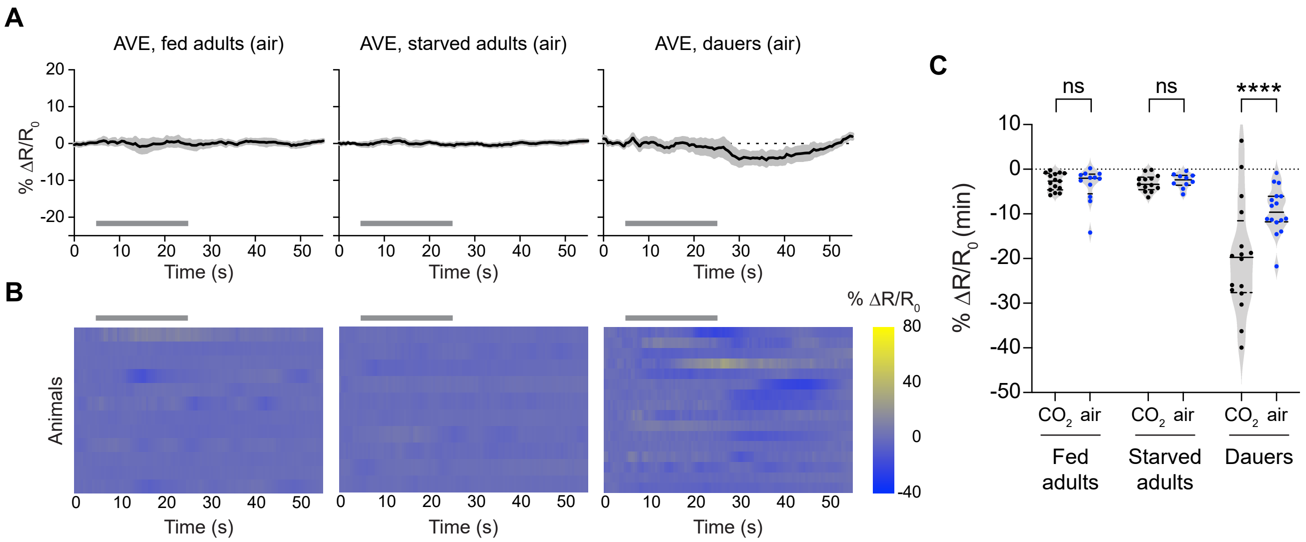

Fig. S4. Inhibitory responses in the AVE interneurons of dauers are evoked by CO_2. (A) Calcium responses in AVE neurons in well-fed adults, starved adults, and dauers to an air control. Solid lines indicate mean calcium responses and shading indicates SEM. Gray bars indicate timing and duration of the CO_2 pulse. Calcium responses were measured using the ratiometric calcium indicator yellow cameleon YC3.60. Responses are to 21% O_2. **(B)** Heatmaps of AVE calcium responses. Each row represents the response of an individual animal. Gray bars indicate the timing and duration of the CO_2 pulse. Response magnitudes in the heatmaps are color-coded according to the scale (% $\Delta R/R_0$) shown to the right. Responses are ordered by hierarchical cluster analysis. **(C)** Quantification of the minimum calcium responses of AVE in well-fed adults, starved adults, and dauers to CO_2 vs. air. Only dauers show CO_2-evoked inhibitory activity in AVE. Each data point indicates the response of a single animal. Solid lines in violin plots show medians and dotted lines show interquartile ranges. ****$p<0.0001$, ns = not significant ($p>0.4$), two-way ANOVA with Sidak's post-test. n = 11-16 animals per life stage and condition.

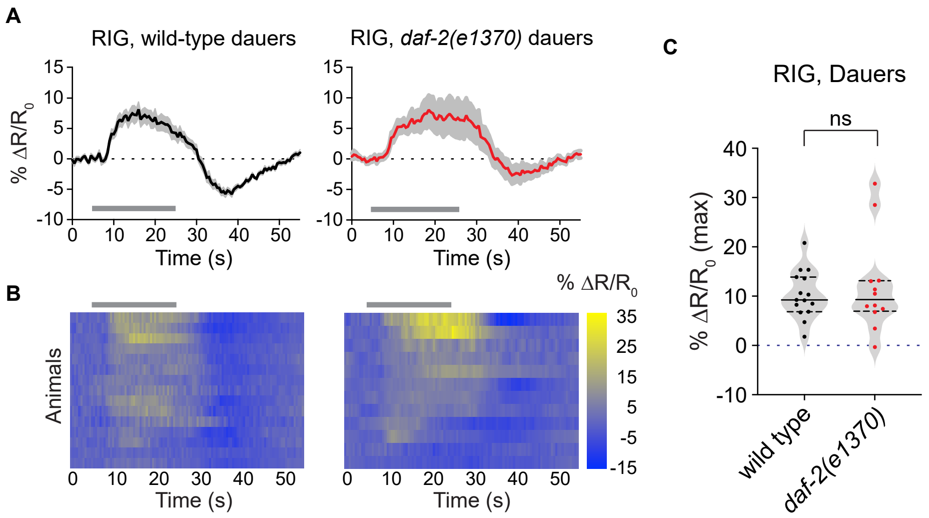

Fig. S5. RIG activity in dauers is not dependent on *daf-2*. (A) CO_2-evoked calcium responses of the RIG neurons of wild-type and *daf-2(e1370)* dauers. Solid lines represent mean calcium responses; shading represents SEM. Gray bars indicate the timing and duration of the CO_2 pulse. Calcium responses were measured using the ratiometric calcium indicator yellow cameleon YC3.60. Responses are to 15% CO_2. **(B)** Heatmaps of RIG calcium responses. Each row represents the response of an individual animal. Gray bars indicate the timing and duration of the CO_2 pulse. Response magnitudes in the heatmaps are color-coded according to the scales (% $\Delta R/R_0$) shown to the right. Responses are ordered by hierarchical cluster analysis. **(C)** Quantification of the maximum responses of RIG in wild-type and *daf-2(e1370)* mutant dauers. ns = not significant (p = 0.6156), Welch's t-test. n = 12-15 animals per genotype. Each data point indicates the response of a single animal. Solid lines in violin plots show medians and dotted lines show interquartile ranges.

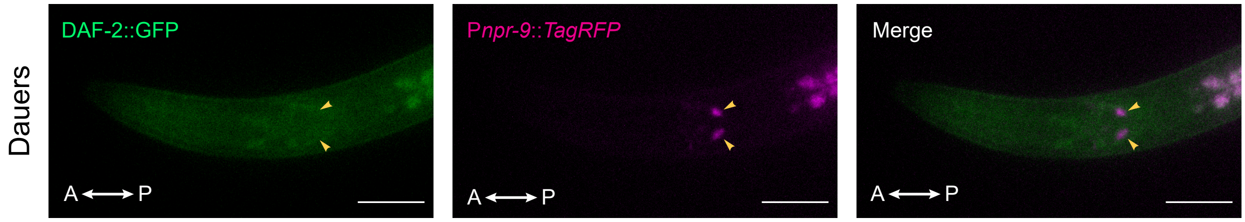

Fig. S6. DAF-2 is not expressed in the AIB neurons of dauers. Epifluorescent images of transgenic dauers co-expressing an endogenously GFP-tagged DAF-2 protein (DAF-2::GFP) and the fluorescent marker TagRFP specifically in AIB (P*npr-9::TagRFP*). Arrowheads indicate the positions of the AIB cell bodies. Scale bars indicate 25 μm.

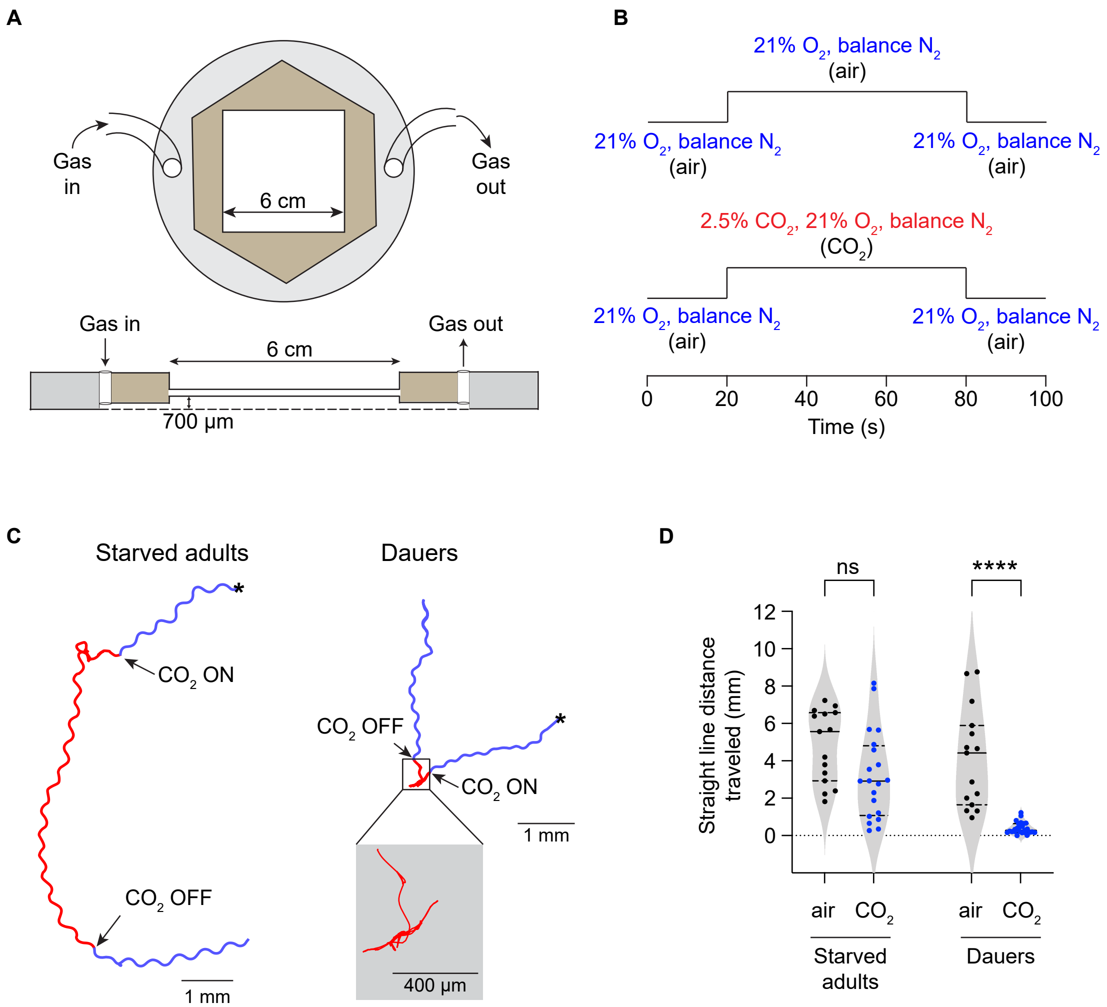

Fig. S7. Behavioral responses to acute increases in CO_2 in starved adults vs. dauers. (**A**) Schematic of the chamber used to measure acute responses to CO_2. Adapted from Rojo Romanos *et al.*, 2018 (13). Animals were placed within the 6 cm arena and exposed to defined gas mixtures while their behavioral responses were recorded. (**B**) Animals were exposed to gas pulse trains of either air (20 s) – CO_2 (60 s) – air (20 s) or air (20 s) – air (60 s) – air (20 s). (**C**) Starved adults and dauers show distinct CO_2-evoked motor behaviors. Representative movement trajectories of a starved adult and a dauer in response to CO_2. Asterisks indicate the position of the animals at the start of videorecording. Blue and red lines indicate movement tracks in response to 20 s exposures to air (21% O_2, balance N_2) and a 60 s exposure to CO_2 (2.5% CO_2, 21% O_2, balance N_2), respectively. (**D**) Straight line distances traveled by starved adults and dauers during a 60 s exposure to either air or CO_2. Each data point indicates distance traveled by a single animal. Solid lines in violin plots show medians and dotted lines show interquartile ranges. ****$p<0.0001$, two-way ANOVA with Sidak's post-test. ns = not significant ($p = 0.1067$).

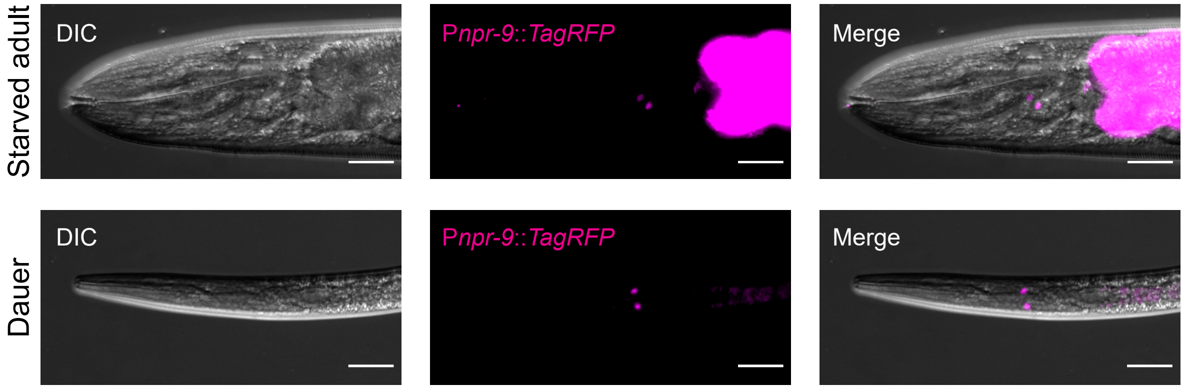

Fig. S8. The AIB-specific promoter shows a similar expression pattern in starved adults and dauers. Epifluorescent images of transgenic starved adults and dauers expressing TagRFP in AIB (P*npr-9::TagRFP*). The bright intestinal fluorescence in starved adults indicates autofluorescence. Scale bars indicate 25 μm.

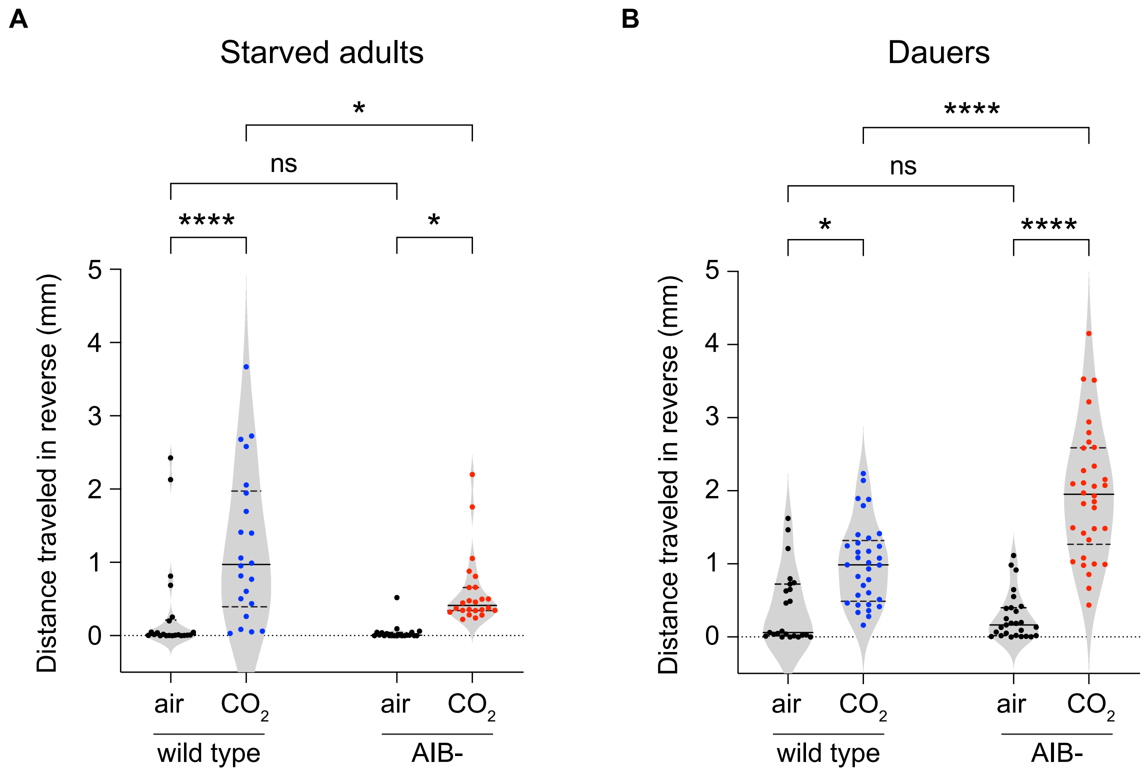

Fig. S9. The AIB neurons differentially regulate CO_2-evoked reversal behavior in starved adults vs. dauers. (A) AIB-ablated starved adults travel significantly less distance in reverse in response to CO_2 compared to wild-type starved adults. Each data point indicates the distance traveled by a single animal during a 60 s exposure to either air or CO_2. Solid lines in violin plots show medians and dotted lines show interquartile ranges. ****$p<0.0001$, *$p<0.05$, two-way ANOVA with Sidak's post-test. n = 21-24 animals per genotype. **(B)** AIB-ablated dauers travel a significantly greater distance in reverse in response to CO_2 compared to wild-type dauers. Conditions and conventions are as in panel A. ****$p<0.0001$, *$p<0.05$, two-way ANOVA with Sidak's post-test. n = 23-34 animals per genotype.

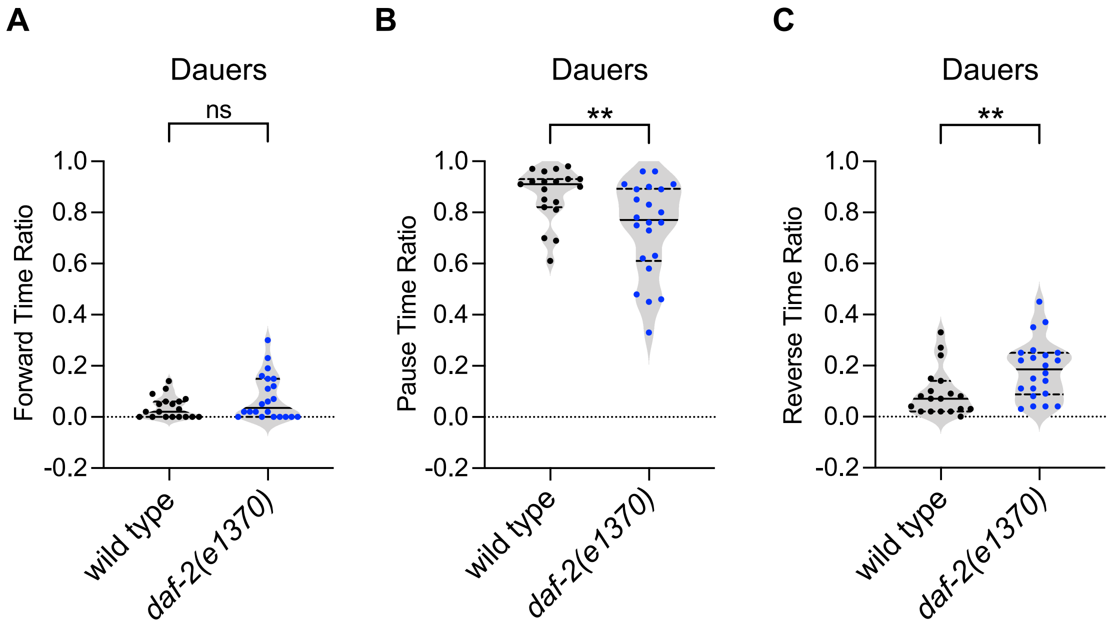

Fig. S10. CO_2-evoked motor output in dauers is dependent on DAF-2 function. (A) Both wild-type and *daf-2(e1370)* mutant dauers show similar forward movement duration in response to CO_2. **(B)** *daf-2(e1370)* dauers show a decrease in CO_2-evoked pause duration compared to wild-type dauers. **(C)** *daf-2(e1370)* dauers show an increase in CO_2-evoked reverse duration compared to wild-type dauers. For A-C, n = 19-22 animals per genotype. Each data point indicates the behavioral response of a single animal. Solid lines in violin plots show medians and dotted lines show interquartile ranges. ***$p<0.01$, Mann-Whitney test; ns = not significant ($p>0.05$), Welch's t-test.

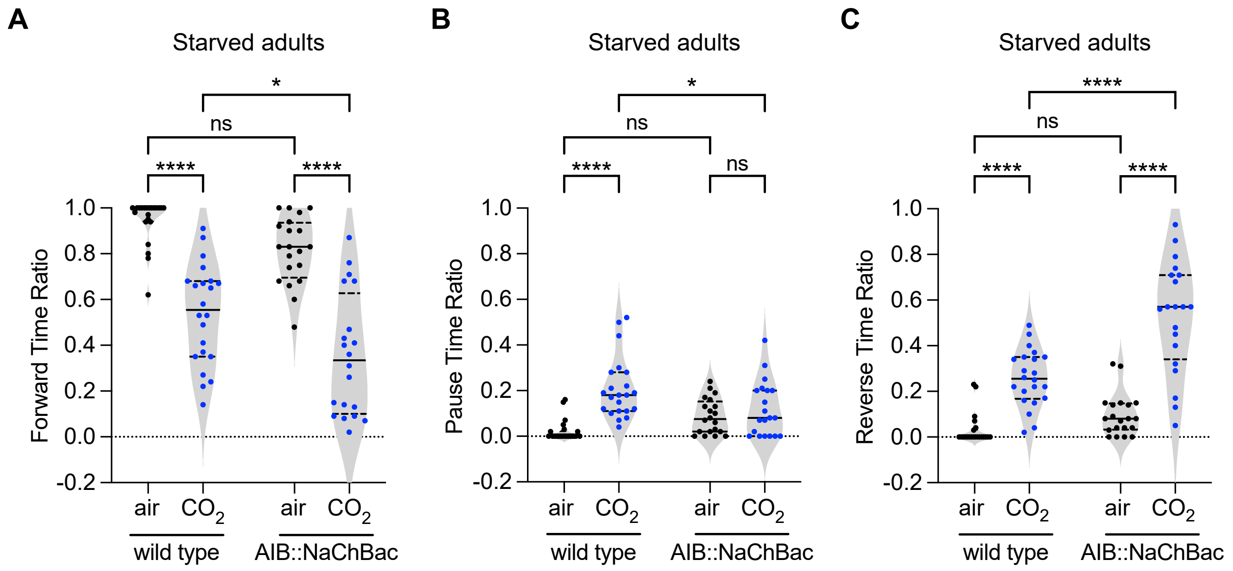

Fig. S11. Increasing AIB excitability in starved adults is not sufficient to mimic dauer-like CO_2-evoked behavior. (A) Both wild-type and AIB::NaChBac starved adults show a reduction in forward movement duration in response to CO_2, but this effect is more pronounced in AIB::NaChBac starved adults. **(B)** Wild-type starved adults but not AIB::NaChBac starved adults increase their pause duration in response to CO_2. **(C)** Both wild-type and AIB::NaChBac starved adults show an increase in reverse movement duration in response to CO_2, but this effect is more pronounced in AIB::NaChBac starved adults. For A-C, n = 20-22 animals per genotype and condition. Each data point indicates the behavioral response of a single animal. Solid lines in violin plots show medians and dotted lines show interquartile ranges. ****$p<0.0001$, *$p<0.05$, ns = not significant ($p>0.1$), two-way ANOVA with Sidak's post-test.

Table S1. List of strains. *lf* = loss-of-function mutation; *ts* = temperature-sensitive.

**Strain**	**Genotype**	**Strain description**	**Source and References**
N2	Wild-type Bristol	wild type	CGC
AX2073	*lin-15(n765ts); dbEx[*P*flp-17::YC3.60, lin-15(+)]*	Cameleon in BAG	de Bono lab (15)
EAH67	*bruEx49[*P*gcy-9::GFP,* P*pax-2::GFP]*	GFP expression in BAG	Hallem lab (16)
PS6028	*syEx1134[*P*twk-3::YC3.60;* P*pax-2::GFP]*	Cameleon in RIG	Sternberg lab (3)
EAH148	*kyIs536[*P*flp-17::p17::SL2::GFP,* P*elt-2::mCherry]; kyIs538[*P*glb-5::p12::SL2::GFP,* P*elt-2::mCherry]; syEx1134[*P*twk-3::YC3.60]*	Cameleon in RIG in BAG-ablated background	Hallem lab (3)
IK1405	*njEx568[*P*ttx-3::YC3.60,* P*ges-1::NLS-RFP]*	Cameleon in AIY	Mori lab (3)
EAH259	*Ex[*P*odr-2(1b)::YC3.60;* P*lin-44::GFP]*	Cameleon in AIB	Hirotsu lab (17)
PS5932	*lin-15(n765); syEx1111[*P*opt-3::YC3.60, lin-15(+)]*	Cameleon in AVE	Sternberg lab (this paper)
EAH381	*che-7(ok2373)*; *Ex[*P*odr-2(1b)::YC3.60;* P*lin44::GFP]*	Cameleon in AIB in *che-7(lf)* background	Hallem lab (this paper)
EAH383	*daf-2(e1370)*; *Ex[*P*odr-2(1b)::YC3.60; lin-44::GFP]*	Cameleon in AIB in *daf-2(lf)* background	Hallem lab (this paper)
EAH382	*daf-2(e1370)*; *syEx1134[*P*twk-3::YC3.60,* P*pax-2::GFP]*	Cameleon in RIG in *daf-2(lf)* background	Hallem lab (this paper)
EAH409	*daf-2(cv20[daf-2::GFP]); otIs643[*P*npr-9::TagRFP]*	*daf-2* endogenous locus tagged with GFP + an AIB marker	Hallem lab (this paper)
OH13918	*otIs643[*P*npr-9::TagRFP]*	AIB marker	Hobert lab (10)
EAH 202	*peIs578[*P*npr-9::casp1,* P*npr-9::Venus,* P*unc-122::mCherry]*	AIB ablation	Iino lab (18)
PS7627	*syIs447[npr-9p::GAL4::VP64::let-858 3'UTR] syIs337[15xUAS::Δpes-10::GFP-let-858 3'UTR]; syIs446[UAS::NaChBac::GFP::let-858 3' UTR]*	NaChBac expression in AIB	C. Chai (Sternberg lab)

Dataset S1. Raw data used in this study and corresponding statistical analysis. Additional files are available on GitHub (https://github.com/HallemLab/Banerjee_et_al_2023).

SI References

1. Stiernagle T (2006) Maintenance of *C. elegans*. In WormBook, www.wormbook.org, 1-11.
2. Scott K (2011) Out of thin air: sensory detection of oxygen and carbon dioxide. *Neuron* 69:194-202.
3. Guillermin ML, Carrillo MA, & Hallem EA (2017) A single set of interneurons drives opposite behaviors in *C. elegans*. *Curr Biol* 27:2630-2639.
4. Rengarajan S, Yankura KA, Guillermin ML, Fung W, & Hallem EA (2019) Feeding state sculpts a circuit for sensory valence in *Caenorhabditis elegans*. *Proc Natl Acad Sci USA* 116:1776-1781.
5. Campbell JC, Chin-Sang ID, & Bendena WG (2017) A *Caenorhabditis elegans* nutritional-status based copper aversion assay. *J Vis Exp* 125:e55939.
6. Karp X (2016) Working with dauer larvae. In WormBook, www.wormbook.org, 1-19.
7. Carrillo MA, Guillermin ML, Rengarajan S, Okubo R, & Hallem EA (2013) O_2-sensing neurons control CO_2 response in *C. elegans*. *J Neurosci* 33:9675-9683.
8. Nagai T, Yamada S, Tominaga T, Ichikawa M, & Miyawaki A (2004) Expanded dynamic range of fluorescent indicators for Ca^{2+} by circularly permuted yellow fluorescent proteins. *Proc Natl Acad Sci USA* 101:10554-10559.
9. Colon-Ramos DA, Margeta MA, & Shen K (2007) Glia promote local synaptogenesis through UNC-6 (netrin) signaling in *C. elegans*. *Science* 318:103-106.
10. Bhattacharya A, Aghayeva U, Berghoff EG, & Hobert O (2019) Plasticity of the electrical connectome of *C. elegans*. *Cell* 176:1174-1189.
11. Babicki S*, et al.* (2016) Heatmapper: web-enabled heat mapping for all. *Nucleic Acids Res* 44:W147-W153.
12. Schindelin J*, et al.* (2012) Fiji: an open-source platform for biological-image analysis. *Nat Methods* 9:676-682.
13. Rojo Romanos T, Ng L, & Pocock R (2018) Behavioral assays to study oxygen and carbon dioxide sensing in *Caenorhabditis elegans*. *Bio Protoc* 8:e2679.
14. Faul F, Erdfelder E, Lang AG, & Buchner A (2007) G*Power 3: a flexible statistical power analysis program for the social, behavioral, and biomedical sciences. *Behav Res Methods* 39:175-191.
15. Bretscher AJ*, et al.* (2011) Temperature, oxygen, and salt-sensing neurons in *C. elegans* are carbon dioxide sensors that control avoidance behavior. *Neuron* 69:1099-1113.
16. Guillermin ML, Castelletto ML, & Hallem EA (2011) Differentiation of carbon dioxide-sensing neurons in *Caenorhabditis elegans* requires the ETS-5 transcription factor. *Genetics* 189:1327-1339.
17. Uozumi T*, et al.* (2012) Temporally-regulated quick activation and inactivation of Ras is important for olfactory behaviour. *Sci Rep* 2:500.
18. Kunitomo H*, et al.* (2013) Concentration memory-dependent synaptic plasticity of a taste circuit regulates salt concentration chemotaxis in *Caenorhabditis elegans*. *Nat Commun* 4:2210.
